# Molecular crowding enhances facilitated diffusion of two human DNA glycosylases

**DOI:** 10.1093/nar/gkv301

**Published:** 2015-04-06

**Authors:** Shannen L. Cravens, Joseph D. Schonhoft, Meng M. Rowland, Alyssa A. Rodriguez, Breeana G. Anderson, James T. Stivers

**Affiliations:** 1 Department of Pharmacology and Molecular Sciences, The Johns Hopkins University School of Medicine, 725 North Wolfe Street, Baltimore, MD 21205-2185, USA; 2 Department of Chemistry and Biochemistry, The University of San Diego, 5998 Alcalá Park, San Diego, CA 92110, USA

## Abstract

Intracellular space is at a premium due to the high concentrations of biomolecules and is expected to have a fundamental effect on how large macromolecules move in the cell. Here, we report that crowded solutions promote intramolecular DNA translocation by two human DNA repair glycosylases. The crowding effect increases both the efficiency and average distance of DNA chain translocation by hindering escape of the enzymes to bulk solution. The increased contact time with the DNA chain provides for redundant damage patrolling within individual DNA chains at the expense of slowing the overall rate of damaged base removal from a population of molecules. The significant biological implication is that a crowded cellular environment could influence the mechanism of damage recognition as much as any property of the enzyme or DNA.

A significant triumph in biochemistry over the last 20 years was the ability to isolate human DNA repair enzymes and study their *in vitro* properties using defined DNA substrates containing damaged sites. Typically, these studies have been performed using dilute conditions, where the concentration of the enzyme, DNA and buffer components were low compared to the concentration of water. Although a wealth of insights into the thermodynamic, kinetic and structural properties of enzymes have resulted from such approaches (1–7), DNA repair enzymes act in a crowded cellular environment with quite different physical properties (8,9). Thus, an open question is how the complex intracellular milieu affects the ability of enzymes to locate and repair damage sites embedded in a large polymeric DNA substrate.

The human intracellular environment has numerous physical properties that could dramatically affect enzyme activity. These include high inorganic ion and metabolite concentrations (10,11), lower dielectric properties (12–14), higher bulk viscosity (15,16), and the presence of high concentrations of macromolecules which consume available volume (‘molecular crowding’) (17,18). Indeed, the concentration of macromolecules in human cells is an astounding ∼100–300 mg/ml (9,19), which means that 10–40% of the total cellular volume is consumed by large molecules (often called the *excluded volume*). Taken together,­ these parameters could affect association of an enzyme with its target in complex ways. For instance, high ion concentrations are expected to shield electrostatic interactions between an enzyme and its highly charged DNA substrate (10,20,21), while a lower dielectric constant could have an opposite effect. Increases in macroscopic viscosity will slow the translational movement of macromolecules and due to entropic effects, crowded environments will push macromolecular association when the complex consumes a smaller volume than the free component species (9,22,23).

Although volume exclusion largely explains the effects of crowded environments on binding equilibria, crowding has been reported to have a surprisingly small effect on the diffusion-controlled association kinetics of macromolecules (24). Indeed, it has been observed that some diffusion-controlled association reactions occur at nearly the same rates in crowded solutions and in cells as they do in dilute solution (24,25). These kinetic effects are counterintuitive, but can be understood by considering that macromolecular crowders alter the macroscopic viscosity and available volume in crowded solutions, but do not change the microscopic viscosity (26,27). Thus, over short nanometer distances, the rotational and translational diffusion of proteins is not greatly affected by crowding because the protein only feels the microscopic viscosity of the solvent that is present in the spaces between the larger crowding molecules (28). Over larger distances, hard sphere repulsion between the protein and crowding molecules increases the effective viscosity and slows translational diffusion (8,28,29). When two binding partners approach one another, they are captured within a low viscosity (high mobility) cage created by the larger crowding molecules, which increases the probability for a productive encounter event. Surprisingly, the capture of two binding partners within a high mobility cage can in some cases offset all of the negative effects of high viscosity on the overall association rate (29).

The above considerations raise the interesting question of what effect molecular crowding has on enzyme association with DNA, and in particular, the property of facilitated diffusion along a DNA chain? Facilitated diffusion on the DNA chain (‘translocation’) is a distinct process that involves transient states of an enzyme and DNA that are not directly observable in equilibrium binding, steady-state or rapid kinetic measurements (1–4,30). Here, we measure the effect of inert crowding agents on the probability that the DNA repair enzymes uracil and 8-oxguanine DNA repair glycosylase will successfully translocate between two damaged sites in a DNA chain. We find that crowding increases the likelihood that each enzyme will successfully translocate between their respective target sites without dissociation to bulk solution and also increases the average translocation distance. For both enzymes, crowding biases the damage search process toward a chain tracking search mode rather than a 3D search mode. Such a crowder-induced transition in the search mode could significantly impact the effectiveness of the damage search in a crowded nuclear environment. These enzymes represent two of the largest superfamilies of glycosylases and their similar behavior in these studies suggests that the findings will be general for other related glycosylases.

## MATERIALS AND METHODS

### General

All experiments with hUNG were performed in a buffer consisting of 20 mM 4-(2-hydroxyethyl)-1-piperazineethanesulfonic acid (HEPES) pH 7.5, 3 mM ethylenediaminetetraacetic acid (EDTA), 1 mM dithiothreitol (DTT), 0.002% Brij 35. This buffer contained a total of 22 mM Na^+^ originating from pH adjustment of the HEPES and EDTA stock solutions. All experiments with hOGG1 were performed in buffer containing 20 mM TrisCl pH 8.0, 1 mM EDTA, and 1 mM DTT, 17 mM NaCl, and 0.1 mg/ml bovine serum albumin (BSA). The catalytic domain of recombinant hUNG was purified as previously described (10) and a detailed description of recombinant hOGG1 purification can be found in Supplemental Methods. The specific DNA (D^S^) containing a non-hydrolyzable deoxyuridine analogue (2′-β-fluoro-2-deoxyuridine) was synthesized in-house as the phosphoramidite form and incorporated during solid phase DNA synthesis as previously described (10). All other oligonucleotides were purchased from either Integrated DNA Technologies or Eurofin and purified in-house by denaturing polyacrylamide gel electrophoresis (PAGE). All DNA sequences are listed in Supplemental Methods. Ethylene glycol, PEG 600, PEG 1500, PEG 3350 and PEG 8K were purchased from Sigma Chemical. To remove ultraviolet (UV) absorbing impurities, these reagents were purified by overnight treatment with activated carbon (0.1 g/ml) and filtered. Dextran 25K and Ficoll 70 were purchased from GE Healthcare.

### Site transfer assay

Site transfer assays were performed as previously reported (30) and the general procedure is recapitulated here. To initiate the reactions, hUNG (20–900 pM) was mixed with a dual uracil ^32^P labeled DNA substrate (40 nM) at 37°C in the presence and absence of varying amounts of PEG 8K, 3350, 1500, 600, ethylene glycol and hemoglobin. At the indicated times, aliquots of the reaction mixture were quenched with Uracil DNA Glycosylase Inhibitor protein (UGI, New England Biolabs) and the abasic sites were cleaved by heating in the presence of ethylene diamine. For hUNG experiments using hemoglobin, the same procedure was followed with the following exceptions: (i) aliquots were quenched in 28 ul of formamide buffer and (ii) abasic sites were cleaved by heating at 95°C for 90 min. For experiments using hOGG1, 1 nM of enzyme was added to 20 nM ^32^P-labeled S20^oG^ in the presence and absence of 20% PEG 8K and 5% hemoglobin. At the denoted times, aliquots of the reaction were quenched with 20 μl formamide loading buffer and heated for 10 min at 95°C. For both enzymes, the discrete DNA fragments generate by heating were resolved by electrophoresis on a denaturing 10% PAGE gel containing 7 M urea. All gels were dried, exposed overnight to a storage phosphor screen and imaged with a Typhoon 8600 phosphorimager (GE Healthcare). All gel images were quantified using QuantityOne (Bio-Rad) by the box method. The time independent overall site transfer probability (*P*_trans_) was calculated using Equation (1) by linear extrapolation of the observed transfer probabilities (*P*_trans_^obs^) to zero time. For experiments involving the use of uracil trap for hUNG, an aliquot of a 10 mM uracil stock solution in water was dried and then reconstituted in reaction buffer such that the final reaction concentration was 10 mM. For experiments involving the use of the 2-amino-6-chloropurine trap (Sigma–Aldrich) trap for hOGG1, a final concentration of 3 mM was added to each reaction from a stock solution prepared in dimethyl sulfoxide (DMSO). Reactions were carried out in 15% DMSO to ensure solubility of the trap. We have shown previously that DMSO does not alter translocation of hOGG1 on DNA (31). Controls established that transfer probabilities in the presence of the hUNG trap (10 and 20 mM) and hOGG1 trap (1 and 3 mM) were independent of trap concentration in the presence and absence of 20% PEG 8K. The intramolecular transfer assay was then performed as described above.

(1) \begin{equation*} P_{{\rm trans}} = \frac{{[{\rm A}]^0 + [{\rm C}]^0 - [{\rm AB}]^0 - [{\rm BC}]^0 }}{{[{\rm A}]^0 + [{\rm C}]^0 + [{\rm AB}]^0 + [{\rm BC}]^0 }} \end{equation*}

### hUNG stopped flow measurements

To measure the association and dissociation rates of a specific site, a 19mer duplex DNA substrate (D^S^) was used that contained a non-hydrolyzable analog of uracil (U^β^ = 2′-β-fluoro-2-deoxyuridine) adjacent to the environmentally sensitive reporter base 2-aminopurine (2-AP) (32). Association rates were measured under second-order conditions by mixing 400 nM D^S^ with 400 nM of hUNG in equal volumes using an Applied Photophysics 720 stopped-flow device in two-syringe mode. The 2-AP fluorescent signal was recorded using a 360 nm long-pass emission filter and a 310 nm excitation wavelength at a temperature of 20°C. Higher temperatures could not be used because the association rates exceeded the time resolution of the instrument. These experiments were conducted in the presence and absence of varying amounts (w/v) of PEG 8K (5, 10, 15, 20%), PEG 600 (5, 10, 20, 30%), and ethylene glycol (20, 40%).

Since the concentrations of each species were equal ([DNA]_0_ = [hUNG]_0_) and much greater than the *K*_D_ for the interaction at a specific site (*K*_D_ = 0.3 nM, Supplementary Table S6), the rate of association (*k*_on_) was determined by fitting the fluorescent traces to Equation (2), where *Y*_0_ is the initial fluorescence intensity (33).

(2) \begin{equation*} {Y}({t}) = {Y}_{\rm f} - {Y}_0 \left( {1 - \frac{1}{{k_{{\rm on}} [{\rm P}] + 1}}} \right) \end{equation*}

To measure dissociation rates, equal volumes of a pre-incubated solution of hUNG and D^S^ (800 and 200 nM final concentrations) were rapid mixed with a trap solution containing 5 μM of a 19mer DNA with a high affinity tetrahydrofuran abasic site mimic (ϕDNA). This trap ensures irreversible trapping of the dissociated enzyme molecules. The kinetic constants were the same when either 5 or 10 μM of the trap was used, establishing that trapping was not rate-limiting. These experiments were conducted in the presence and absence of varying amounts (w/v) of PEG 8K (5, 10, 15, 20%). The resultant curves were fit to either a single or a double exponential decay equation as required (see ‘Results’ section).

### hUNG steady-state kinetic measurements

Equal volumes of hUNG (30 pM final concentration) and a 5′ ^32^P-labeled 90mer DNA substrate containing a single uracil (1U^90^) (1–32 nM final concentration) were mixed in the standard buffer at 37°C. At various times, 4 μl portions were removed and quenched with 4 μl UGI (0.2 units/μl). Sample treatment and gel procedures were identical to what is described above for the site transfer assay. The same procedure was used to determine the steady-state kinetic parameters of the 5′ ^32^P-labeled 30mer DNA substrate containing a single uracil (1U^30^) (10–400 nM final concentration) at 25°C. Initial rates for both substrates were determined by linear regression using plots of product concentration against time and the software program Prism (Graphpad Software, Inc.). The experiments were performed in the presence and absence of 20% (w/v) PEG 8K.

The *k*_cat_ values for 1U^90^ and 1U^30^ at 75 mM total cation concentration in the presence and absence of 20% PEG 8K were measured essentially as described above for 1U^90^. The 75 mM total cation concentration was established by adding 53 mM KGlu to the standard reaction buffer and gels for separating the products derived from 5′ FAM-labeled 1U^30^ were imaged with a Typhoon 8600 phosphorimager (GE Healthcare) without drying. Bands were quantified by histogram volume analysis using QuantityOne (Bio-Rad). The values for *k*_cat_ were obtained by using high substrate concentrations such that the rate was independent of concentration. This condition was confirmed by measuring identical rates at two substrate concentrations in the range 0.4–3 μM. The reported *k*_cat_ values are an average of the rates determined at the two substrate concentrations.

Kinetic measurements on a short hairpin substrate containing 6 U/A pairs within the 11 bp stem region (6U^11^) were performed using a continuous fluorescence assay (34). Fluorescein emission (*λ*_ex_ = 494 nm, *λ*_em_ = 518 nm) was monitored at 10 s time intervals using a SPEX Fluoromax-3 spectrofluorometer at 25°C using excitation and emission slit widths of 1 and 2 nm, respectively. The experiment was performed in the presence and absence of PEG 8K [5, 10 and 20% (w/v)] and ethylene glycol [20 and 40% (w/v)].

### Ion activity measurements

The sodium ion activity in the presence of 20% (w/v) PEG 8K was determined using a Cole-Parmer sodium ion selective electrode (ISE). Standard sodium solutions were made over the range of 2–1000 ppm in the presence and absence of 20% PEG 8K. An ionic strength adjuster (ISA, 4 M NH_4_Cl/4 M NaOH) was added to each standard in a 1:50 dilution and a magnetic stirrer was used to ensure a constant stirring rate during ion potential readings. The potential was recorded for each solution using an Accument AR25 Dual Channel pH/Ion Meter (Fischer Scientific) in mV mode. The electrode was thoroughly rinsed with a 1:50 solution of ISA in distilled water and dried between readings. The slopes of a semi-log plot of the mV reading (linear axis) against the sodium concentration (log axis) were used to determine the sodium ion activity coefficients in aqueous solution and 20% PEG 8K.

## RESULTS

### Crowding effects on DNA translocation

**Figure 1.**
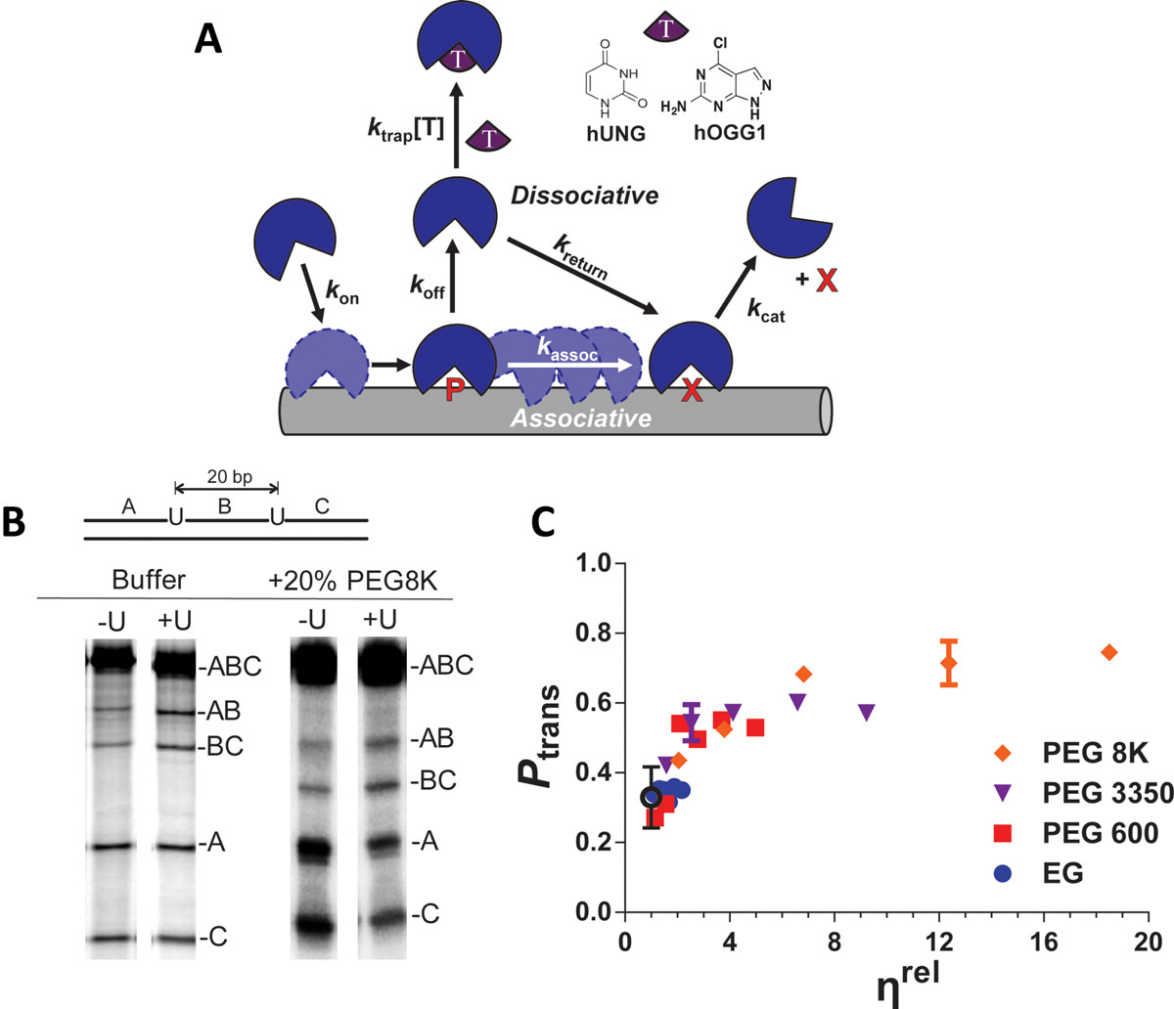
Approach for measuring site transfer probabilities of hUNG and hOGG1. (**a**) The multistep search and repair pathway used by DNA glycosylases. The pathway begins with association to form a nonspecific DNA complex followed by associative and dissociative transfer steps along the DNA chain until a damaged site is located. Site transfer probability measurements involve the use of substrates with two sites of known separation and the determination of the likelihood that an enzyme molecule that produces product (P) at one site successfully transfers to the second site (X) without dissociating to bulk solution (3,30,31). The method has been modified to include a ‘molecular clock’ where a small molecule trap [**T** = U for hUNG, or **I** for hOGG1] is used to prevent transfers by the dissociative pathway, allowing for direct measurement of associative transfers only (see text). (**b**) Phosphorimages of the products derived from reaction of hUNG with a 90mer substrate (S20^U^) with two uracils spaced 20bp apart in dilute buffer and in buffer containing 20% PEG 8K (±U denotes the presence and absence of uracil trap). The increased transfers in the presence of 20% PEG 8K are indicated by the increased levels of the (A) and (C) bands (double excision fragments) as compared to AB and BC bands (single excision fragments). (**c**) The transfer probabilities between uracil sites spaced 20 bp apart (measured at 37°C) as a function of relative viscosity (*η*^rel^ = *η*^crowder^/*η*^buffer^) for a series of PEG polymers. Relative viscosities of these cosolutes were obtained from reference (29). The *P*_trans_ value for buffer alone is indicated by the black open circle.

We first probed the effects of solution viscosity and excluded volume on translocation of hUNG between its target uracil sites in DNA, using our previously described site transfer assay (3,30). Translocation between two uracil sites embedded in a single DNA chain can occur by an *associative* pathway that involves movement of a loosely associated enzyme molecule on the surface of the DNA chain, or a *dissociative* pathway that involves reversible short-range excursions from the DNA surface (Figure 1a). The overall probability of transferring between uracil sites (*P*_trans_) is the sum of the probabilities of transferring by each individual pathway (i.e. *P*_trans_ = *P*_assoc_ + *P*_diss_) and those enzyme molecules that fail to transfer are lost to bulk solution after reacting at only a single site. The contribution of the associative pathway in isolation can be determined by adding a small molecule active site trap to the transfer reaction (**T**, Figure 1a). High concentrations of trap serve to capture all enzyme molecules undergoing dissociative excursions, which selectively blocks this transfer pathway, while leaving the associative pathway intact (30,31). *P*_trans_ is calculated from the relative amounts of DNA product fragments that result from uracil excision at only a single site (fragments AB and BC), and the double site cleavage events that reflect successful intra-site transfer (resulting in fragments A and C). These 5′ and 3′ ^32^P end-labeled DNA fragments are electrophoretically separated on a denaturing polyacrylamide gel and quantified by phosphorimaging as shown in Figure 1b (see ‘Materials and Methods’ section and Equation (1) for further details). It is useful to note that intramolecular site transfer gives rise to more A and C transfer products relative to the AB and BC single site excision products, which can be discerned by simple visual inspection of the band intensities at low extents of reaction (Figure 1b).

We initially explored how *P*_trans_ was affected by 0–30% (w/v) ethylene glycol (EG), PEG 600, 1500, 3350 and 8K using a DNA substrate that contained two uracil sites spaced 20 bp apart (Figure 1b). Varying the polymer size and concentration allowed us to probe both viscosity and crowding effects, with the latter effect expected to become more significant with larger polymers (29,35). Representative data in the presence of 20% PEG 8K are shown in Figure 1b, where a large increase in *P*_trans_ is apparent in the presence and absence of the uracil trap (compare A and C bands in the buffer only and PEG 8K lanes in Figure 1b). *P*_trans_ was found to increase and then plateau as the concentration reached about 15% (w/v), regardless of the PEG polymer that was used (Supplementary Figure S1a). In contrast, the small molecule viscogen EG showed no effect on *P*_trans_ over the concentration range 0–30% (w/v) (Supplementary Figure S1b). It is important to point out that the large increases in *P*_trans_ for PEG polymers excludes the possibility of strong interactions of these polymers with either the DNA or the enzyme (36).

Given that both transfer pathways involve diffusional processes, which should be slowed in a linear fashion by increases in bulk solution viscosity (Figure 1a), we explored whether the transfer probability changed linearly with respect to the relative viscosity of the polymer solutions (*η*^rel^ = *η*^crowd^/*η*^buffer^) (Figure 1c). In this analysis, the relative viscosity values (Supplementary Table S1) were obtained from the literature (29). While *P*_trans_ did initially increase with viscosity in the range *η*^rel^ < 4, it eventually showed downward curvature and reached a plateau level of *P*_trans_ ∼ 0.8. In the presence of 20% PEG 8K, nearly every enzyme molecule that reacted at one site made it to the second site 20 bp away, as compared to only 1 in 3 molecules in dilute buffer (Supplementary Table S3). In contrast, *P*_trans_ was unchanged when the concentration of the small EG viscogen was increased to 30% (w/v) (*η*^rel^ = 2.2) (Supplementary Figure S1b). These findings indicate that viscosity changes alone cannot account for the increases in *P*_trans_, and that the enhancement of *P*_trans_ requires high molecular weight polymers (i.e. crowding).

Control experiments were performed to explore other possible effects of PEG polymers that were unrelated to viscosity and crowding effects. First, we confirmed that other structurally distinct crowding agents (Dextran 25K and Ficoll 70) also increased *P*_trans_ in a concentration dependent fashion and approached the same plateau value at high concentrations and viscosity observed with PEG polymers (Supplementary Figures S1c and S1d). Thus, the general observations are independent of the chemical structure and size of large crowders. We also investigated whether the results might arise from large changes in the Na^+^ ion activity by PEG polymers. However, using a sodium ion-selective electrode, we found that the activity of Na^+^ decreased only minimally upon addition of 20% PEG 8K (49 ± 1 mV·M^−1^ in buffer and 41 ± 3 mV·M^−1^ in 20% PEG 8K) (Methods and Supplementary Table S2). Based on our previous studies on the salt dependence of *P*_trans_ (10), a small ∼15% reduction in the sodium ion activity would not have any significant effect on *P*_trans_ and would have no effect on *P*_assoc_, which is resistant to changes in salt concentration (30).

We point out that the reported *P*_trans_ values are not corrected for the kinetic efficiency (*E*) at which hUNG excises uracil once a site is encountered (30). Thus, the observed *P*_trans_ = *E* × *P*_trans_^true^ reports on the combined effects of crowding arising from viscosity, molecular crowding, as well as any changes in the excision efficiency once the site is reached. In the context of this work, corrections for the excision efficiency changes are superfluous because the excision efficiency in dilute buffer is already large (*E* = 0.81 ± 0.16) (30) and the excision efficiency in the presence of 20% PEG 8K is even larger based on the maximum *P*_trans_ values reported in Supplementary Table S3 (*E* ≥ 0.92). The maximal change in excision efficiency is therefore only 10–11%, which is within experimental error of excision efficiency measurements. Furthermore, the following site spacing studies are performed with a single concentration of PEG 8K, which makes the data entirely independent of any changes in the excision efficiency.

Given the representative behavior of PEG polymers and the large body of useful polymer theory that has focused on PEG (35,37–40), we performed all additional experiments using 20% PEG 8K. The use of 20% PEG 8K as the standard crowding condition allowed us to further probe both viscosity and molecular crowding effects on the site spacing dependence of site transfer. Importantly, the relative viscosity of 20% PEG 8K is nearly 13-fold greater than water (29) and its larger radius of gyration as compared to hUNG would be expected to produce significant volume exclusion effects (29,35).

### Crowding primarily enhances the associative transfer pathway

To investigate the fundamental basis for the observed polymer induced enhancements of *P*_trans_, we made site transfer measurements using substrates with site spacings in the range 5–55 bp in the presence and absence of the uracil trap so that the relative contributions of the associative and dissociative pathways could be determined. Controls established that the observed transfer probabilities in the presence of the uracil trap (10 or 20 mM) were independent of trap concentration even in the presence of 20% PEG 8K (Supplementary Figure S2).

**Figure 2.**
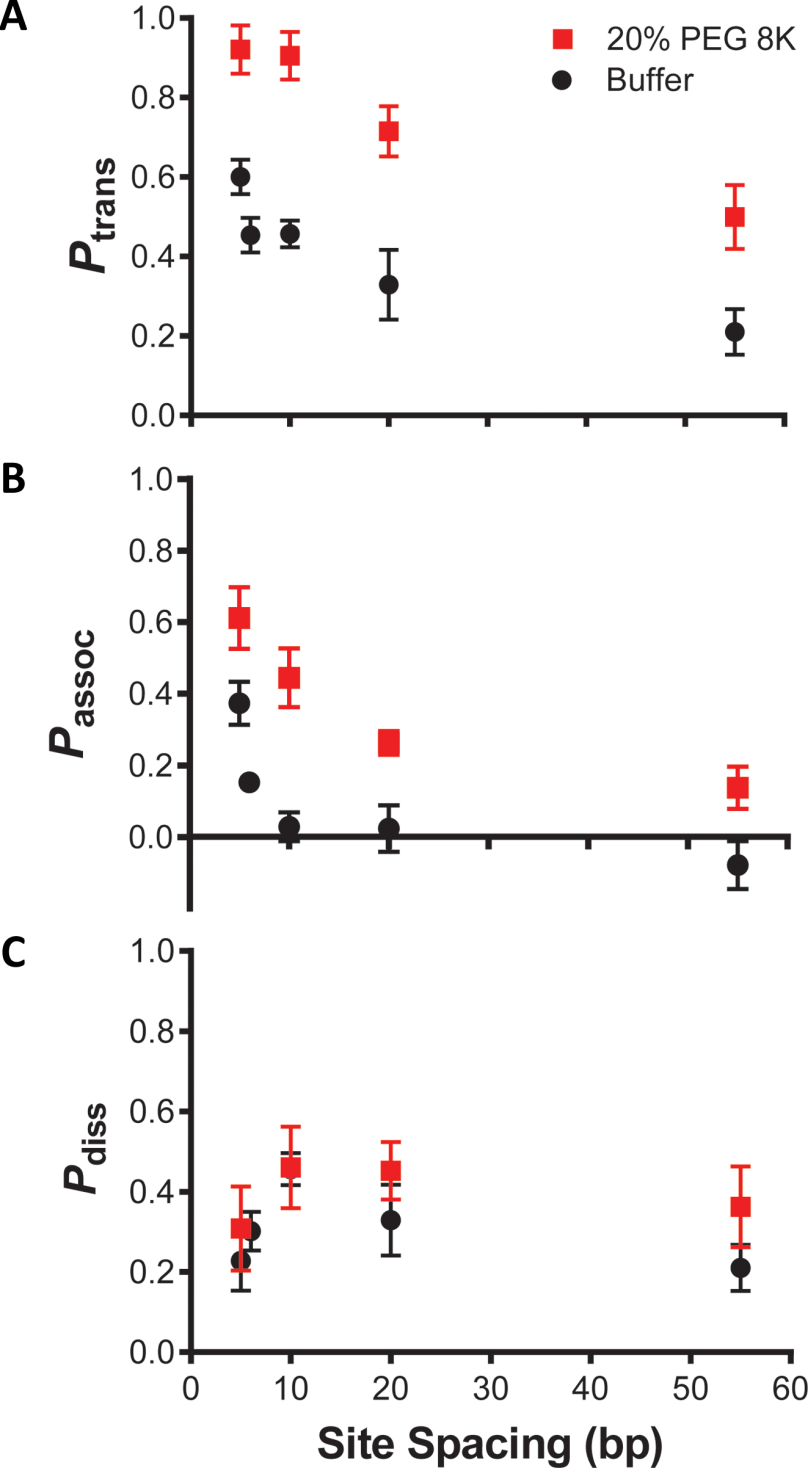
Effect of 20% PEG 8K on the intramolecular site transfer probability between uracil sites of variable spacing. All data obtained from dilute buffer conditions (black) have been previously reported and are displayed here for comparison (30). (**a**) Overall site transfer probabilities (*P*_trans_) as a function of spacing length between uracil sites in the presence (red) and absence (black) of PEG 8K. (**b**) The increased contribution of the associative transfer pathway (*P*_assoc_) and the longer associative transfer distances in the presence of PEG 8K were determined using high concentrations of uracil trap (10 and 20 mM). (**c**) A smaller increase in the probability of dissociative transfers (*P*_diss_ = *P*_trans_ − *P*_assoc_) was observed for all site spacings in the presence of PEG 8K.

In the absence of the uracil trap, the overall site transfer probabilities for all site spacings increased significantly in the presence of 20% PEG 8K (Figure 2a, compare red and black data, see also Supplementary Table S3). The dramatic increases in the transfer probabilities also persisted in the presence of the uracil trap (Figure 2b), indicating that the overall effect was dominated by an increase in associative transfers. Strikingly, introduction of 20% PEG 8K resulted in the persistence of associative transfers for uracils site spacings as large as 55 bp, whereas in dilute buffer solution the associative pathway was abolished for site spacings ≥10 bp (Figure 2b). This indicates that hUNG can traverse a larger linear distance on the DNA chain in the presence of crowding. In contrast, only a small increase in *P*_diss_ was observed in the presence of 20% PEG 8K (Figure 2c).

### Crowding effects on translocation of hOGG1

We were interested in whether the above effects of crowding agents on DNA translocation were specific to hUNG, or alternatively, might reflect a more general property that extended to other DNA glycosylases. Thus we explored the general effects of crowding agents on the site transfer mechanism of hOGG1, a representative enzyme from the large HhH superfamily of DNA glycosylases that removes the oxidized base 8-oxoguanine from DNA (41,42). We have previously determined the site transfer properties of hOGG1 using the same approach as used with hUNG, except that an 8-oxoG analogue (2-amino-6-chloropurine) was used as the small molecule trap (Figure 1a) (31). In initial kinetic experiments using a 31mer DNA duplex containing a single 8-oxoguanine site, we found that 20% PEG 8K reduced the steady-state turnover rate of hOGG1 by ∼8-fold as compared to buffer (Supplementary Figure S3). An inhibitory effect of 20% PEG 8K on the uracil excision rate was also observed with hUNG under *k*_cat_ conditions (see below and Supplementary Table S5). Similar to hUNG, the overall site transfer probability (*P*_trans_) of hOGG1 over a site spacing of 20 bp increased in the presence of 20% PEG 8K (*P*_trans_^buffer^ = 0.34 ± 0.09, *P*_trans_^PEG8K^ = 0.50 ± 0.03).

**Figure 3.**
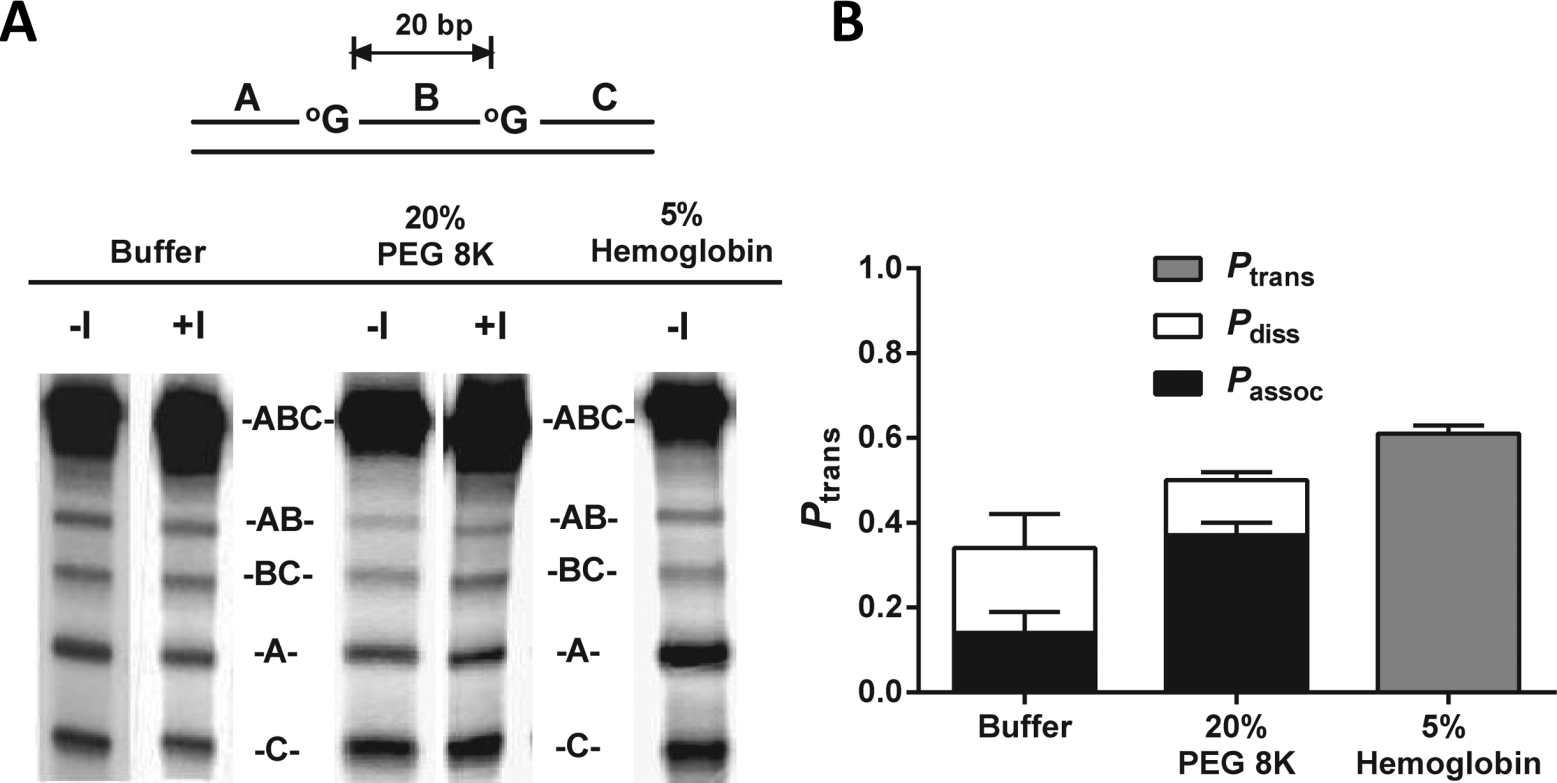
Effect of 20% PEG 8K and 5% hemoglobin on the site transfer probability of hOGG1 over a 20 bp spacing (*P*_trans_). (**a**) Phosphorimages of polyacrylamide gels showing the separation of the reaction products generated from excision of 8-oxoG. The presence or absence of the molecular trap (Figure 1) is indicated by ±I. (**b**) Partitioning of the total transfers between the associative (*P*_assoc_) and dissociative (*P*_diss_) pathways. Error bars show the SD of three replicate determinations of *P*_trans_. Overall *P*_trans_ was found to increase when PEG 8K was added, primarily due to an increase in the relative contribution of the associative pathway. A similar increase in *P*_trans_ was observed upon addition of hemoglobin, though the contributions of *P*_assoc_ and *P*_diss_ could not be discerned due to precipitation upon addition of the trap.

We were curious if the origin of the effect on the overall *P*_trans_ for hOGG1 arose from enhanced probability of transfer by the associative pathway as seen with hUNG (Figure 3). This indeed turned out to be the case as associative transfers of hOGG1 increased by ∼3-fold in the presence of 20% PEG 8K, while change in the probability of dissociative transfers was negligible (Figure 3, Supplementary Table S4). In control experiments we established that the level of associative transfers in the presence of PEG 8K was independent of the trap concentration (Supplementary Figure S2). The fact that the magnitude of the effects on *P*_trans_ and *P*_assoc_ are not as pronounced for hOGG1 as they are for hUNG is consistent with an excluded volume effect. The significance of volume exclusion can be estimated by the size differential between the proteins of interest and the crowding agent (29). Since hOGG1 is larger than hUNG we would predict reduced impact from the crowding agent on hOGG1 translocation.

### Effects of a protein crowder on translocation

The interesting consequences of inert polymer crowders on the translocation of both glycosylases begged the question if the effect can be extended to protein crowders. After a survey of possible protein crowding agents, we chose hemoglobin because it is large enough to exclude significant volume, and it has a favorably low number of ionic groups that helped minimize changes in ionic strength upon addition of high hemoglobin concentrations. In our buffer conditions, hemoglobin was soluble up to 50 mg/ml [5% (w/v)] and this concentration was used in these experiments. Much like 20% PEG 8K, *P*_trans_ for hOGG1 was found to increase in the presence of hemoglobin (Figure 3). The larger increase in *P*_trans_ observed in 5% hemoglobin as compared to 20% PEG 8K bolsters our conclusion that the effect is not due simply to viscosity since the hemoglobin solution is significantly less viscous (43). We could not partition this effect into contributions from associative and dissociative transfers because addition of the trap caused gross precipitation.

Similar experiments using hUNG produced more complex results than with hOGG1. First, 50 mg/ml hemoglobin inhibited the steady-state rate of hUNG by ∼1000-fold, while having only a negligible effect on hOGG1 (Supplementary Figure S3). This significant effect on the reaction rate precluded any definitive conclusions on translocation (Supplementary Figure S4). We provide a reasonable analysis of this data in the Supplemental Discussion, but do not include it in our global conclusions about the effect of crowding on translocation due to the specific inhibition by hemoglobin. The disparities in the effects of hemoglobin on *P*_trans_ and the reaction rates for hUNG and hOGG1 indicate the limitations of using protein crowding agents, which are clearly not inert in every context and have the potential to mask viscosity and molecular crowding effects by interacting with the macromolecules of interest.

### Crowding decreases turnover of hUNG with large DNA substrates

The similar effects of molecular crowding agents on DNA translocation by hUNG and hOGG1 suggests that it is a general mechanism that facilitates DNA chain surveillance by DNA glycosylases. Since the mechanism of facilitated diffusion depends on the thermodynamic and kinetic stability of intermediate states along the search and repair pathway (Figure 1), we sought to dissect the effects of PEG polymers on each microscopic step of the hUNG reaction as described below.

**Figure 4.**
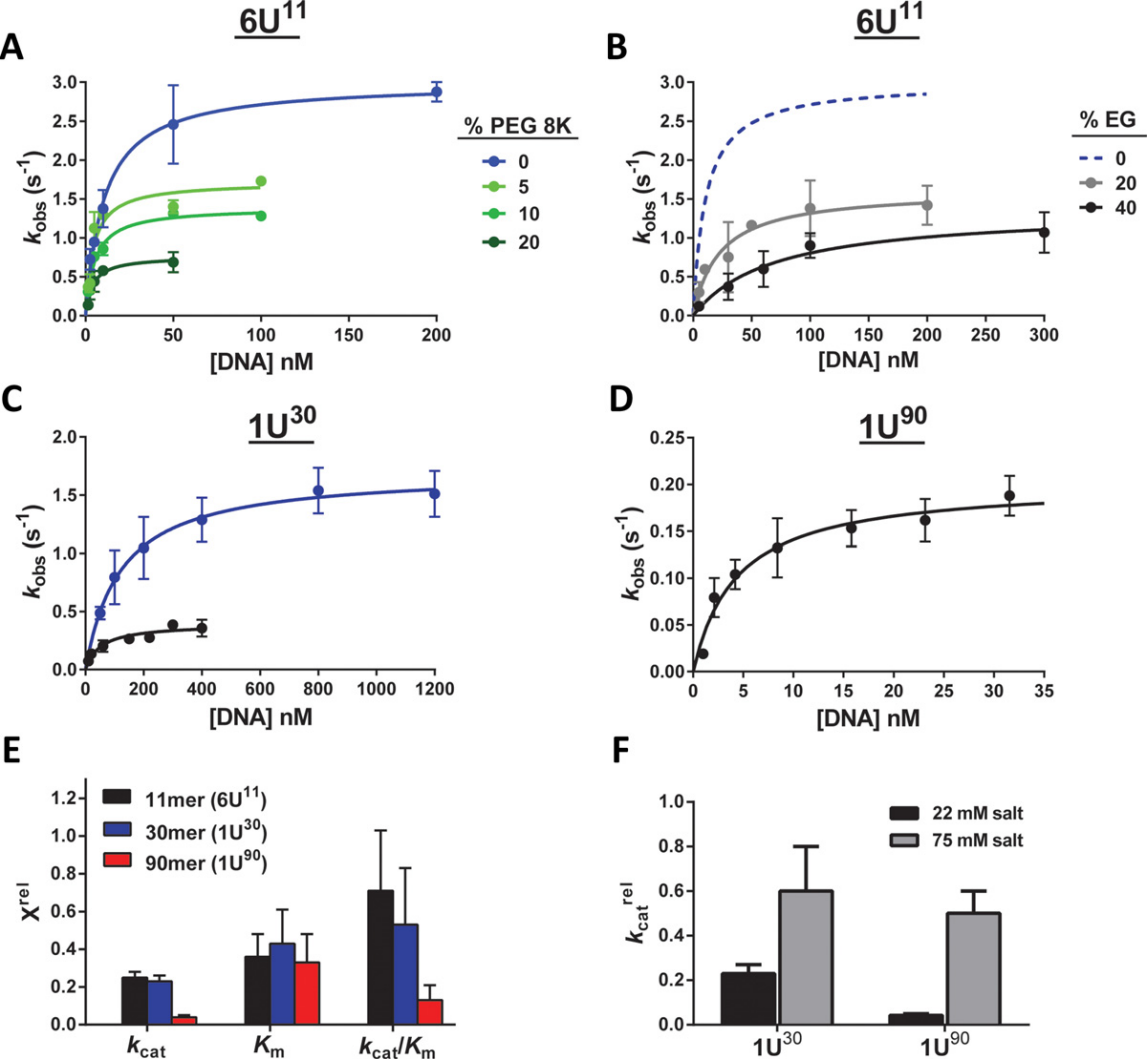
The effect of molecular crowding agents on the steady-state kinetics of hUNG acting on short (11 and 30 bp) and long (90 bp) duplex DNA substrates. The kinetic parameters obtained from all of the data sets in this figure are reported in Supplementary Table S5. (**a**) Effect of 5–20% PEG 8K on the initial rates of reaction of hUNG with a DNA hairpin substrate containing an 11 bp stem (6U^11^). (**b**) Effect of 20–40% EG on the initial reaction rates of hUNG with 6U^11^. For comparison, the dashed blue line is the curve for dilute buffer shown in panel (a). (**c**) Effect of 20% PEG 8K (black) on the initial reaction rates of hUNG with a 30 mer duplex (1U^30^) in comparison to dilute buffer (blue). (**d**) Initial rates of uracil excision from the 90mer duplex (1U^90^) in the presence of 20% PEG 8K. Previously published data with the same substrate in the absence of PEG 8K are reported in Supplementary Table S5. (**e**) The relative effect of 20% PEG 8K on the steady-state kinetic parameters (*X*^rel^) for hUNG acting on short (6U^11^ and 1U^30^) and long (1U^90^) duplex DNA substrates. *X*^rel^ is defined as the kinetic parameter obtained in the presence of PEG 8K divided by that obtained in buffer alone (see Supplementary Table S5). (**f**) The salt-dependent change in *k*_cat_ for 30 bp (1U^30^) and 90 bp (1U^90^) DNA substrates in the presence of molecular crowding agents. The effect of 20% PEG 8K on *k*_cat_ for both substrates was determined in presence of high salt (75 mM total cation concentration, gray bars). For comparison, the black bars show the *k*_cat_ values determined at low salt (22 mM cation concentration, see Supplementary Figure S5). The parameter *k*_cat_^rel^ is defined as the *k*_cat_ value determined in the presence of 20% PEG 8K divided by the value obtained using buffer alone. The *k*_cat_ values for both 1U^30^ and 1U^90^ dropped ∼2-fold upon addition of 20% PEG 8K (1U^30^): *k*_cat_ decreased from 2.1 ± 0.9 to 1.2 ± 0.5 s^−1^; 1U^90^*k*_cat_ decreased from 10.5 ± 0.4 to 5 ± 2 s^−1^. The similar effect of PEG 8K for both substrates at high, but not low salt, supports the proposal that rate-limiting associative transfers limit turnover of the large substrate at low salt. The differences in the absolute *k*_cat_ values for each substrate are due to sequence dependent differences in the steady-state turnover rate (5,49).

We first investigated the effect of increased associative transfers on the steady-state kinetic parameters for uracil excision from short and long DNA substrates (Figure 4a–d). In these studies we used (i) a molecular beacon hairpin DNA construct (6U^11^) that contained six closely spaced uracils within an 11 bp stem, allowing continuous fluorescence measurement of the steady-state rates, (ii) a 30mer with a single central uracil (1U^30^) and (iii) a 90mer duplex with a single central uracil (1U^90^). Taking advantage of the convenient fluorescence assay available with 6U^11^ (see Methods), the *k*_cat_, *K*_m_ and *k*_cat_/*K*_m_ values were determined in the presence of dilute buffer, 5–20% PEG 8K, and 20–40% EG. For 1U^30^ and 1U^90^, the same parameters were measured in discontinuous assays using 5′-^32^P labeled DNA using dilute buffer and a single concentration of 20% PEG 8K. The kinetic parameters for these substrates under the various conditions are summarized in Supplementary Table S5.

The use of EG as a small molecule viscogen resulted in apparent inhibition of hUNG. This effect was manifested most prominently in a 7-fold increase in *K*_m_ and an order-of-magnitude reduction in *k*_cat_/*K*_m_ (Figure 4b, Supplementary Table S5). Such inhibition by EG is not entirely unexpected because the related molecule glycerol has been shown to bind to the UNG active site in the position occupied by the uracil base and inhibit the reaction (44). In addition, inhibitory effects of EG have been observed previously in the context of protein–protein association and have been attributed to preferential hydration of proteins in EG solutions, resulting in slowed association rates due to increased difficulty of stripping away additional water molecules (24).

The most salient findings from the kinetic studies utilizing 20% PEG 8K were that (i) *k*_cat_ and *K*_m_ values for both 6U^11^ and 1U^30^ were decreased by ∼4-fold, such that the *k*_cat_/*K*_m_ values were only slightly lower than dilute buffer, and (ii) 1U^90^ exhibited a much larger 20-fold decrease in *k*_cat_ and a 10-fold decrease in *k*_cat_/*K*_m_ (Figure 4e, Supplementary Table S5). We note that the origin of the apparent inhibitory effect of PEG for the 1U^90^ substrate is almost entirely on *k*_cat_, requiring that it arises from a step involving the ES complex, but not the free enzyme (this is distinct from the inhibition by EG, which primarily binds to free E). Further, the step must occur before or after glycosidic bond cleavage, because the chemical step is very rapid for all of these substrates (*k*_cl_ = 240 s^−1^) (30). Thus, the only fundamental difference between the ES complex for 1U^90^ and the shorter substrates is the presence of additional non-specific flanking DNA.

This viewpoint led us to hypothesize that the slow turnover of 1U^90^ was due to increased time spent by hUNG in repetitive associative and dissociative transfers on non-specific DNA. This effect includes the increased time spent to reach the target site (for protein molecules that associate far from the uracil site), in addition to transfers along the flanking DNA after uracil excision. We tested this proposal by measuring the effect of 20% PEG 8K on the *k*_cat_ values for the 1U^30^ and 1U^90^ substrates in the presence of a higher salt concentration that is known to disrupt non-specific DNA binding (10). The expectation was that the *k*_cat_ effect would be equalized under such conditions if turnover was indeed limited by the time spent in associative transfers on the non-specific DNA sequences within 1U^90^. This expectation was confirmed because the effect of PEG 8K on the *k*_cat_ value of 1U^90^ was reduced to 2-fold, which is indistinguishable from the 2-fold effect seen with 1U^30^ at high-salt (Figure 4f). We conclude that crowding increases the time spent in associative transfers on DNA and that these transfers can greatly reduce steady-state turnover when the DNA chains are long.

### Crowding has little effect on non-specific DNA binding

To understand the basis for the effects of crowding on *P*_trans_, it is useful to interrogate the equilibrium effects on non-specific DNA binding because translocation involves non-specific DNA interactions. To explore this property, binding measurements were made by following the increases in fluorescence anisotropy that accompanied hUNG binding to a 5′-fluorescein end-labeled non-specific 15mer duplex DNA (D^N^) (Supplementary Figure S5). In contrast with the large increase in the associative transfer distances in the presence of 20% PEG 8K (Figure 2b), we measured only a 2-fold increase in the non-specific equilibrium dissociation constant (*K*_D_^N^ = 1.3 ± 0.5 μM in buffer; *K*_D_^N^ = 2.3 ± 0.5 μM in 20% PEG 8K). Thus, the presence of 20% PEG 8K slightly disfavors equilibrium binding.

The different effects of crowding on binding and site transfer indicates that the major ground states involved in equilibrium binding measurements do not share the same DNA interactions as the transient enzyme states involved in site transfer (Figure 1a). This result and interpretation is consistent with previous findings where methylphosphonate backbone substitutions and high salt concentrations produced large increases in *K*_D_^N^, but did not alter *P*_assoc_ (45). Nevertheless, the *K*_D_ places an important thermodynamic constraint on the system that must be compatible with the kinetic and *P*_trans_ measurements.

### Crowding effects on the association rate with a specific site

**Figure 5.**
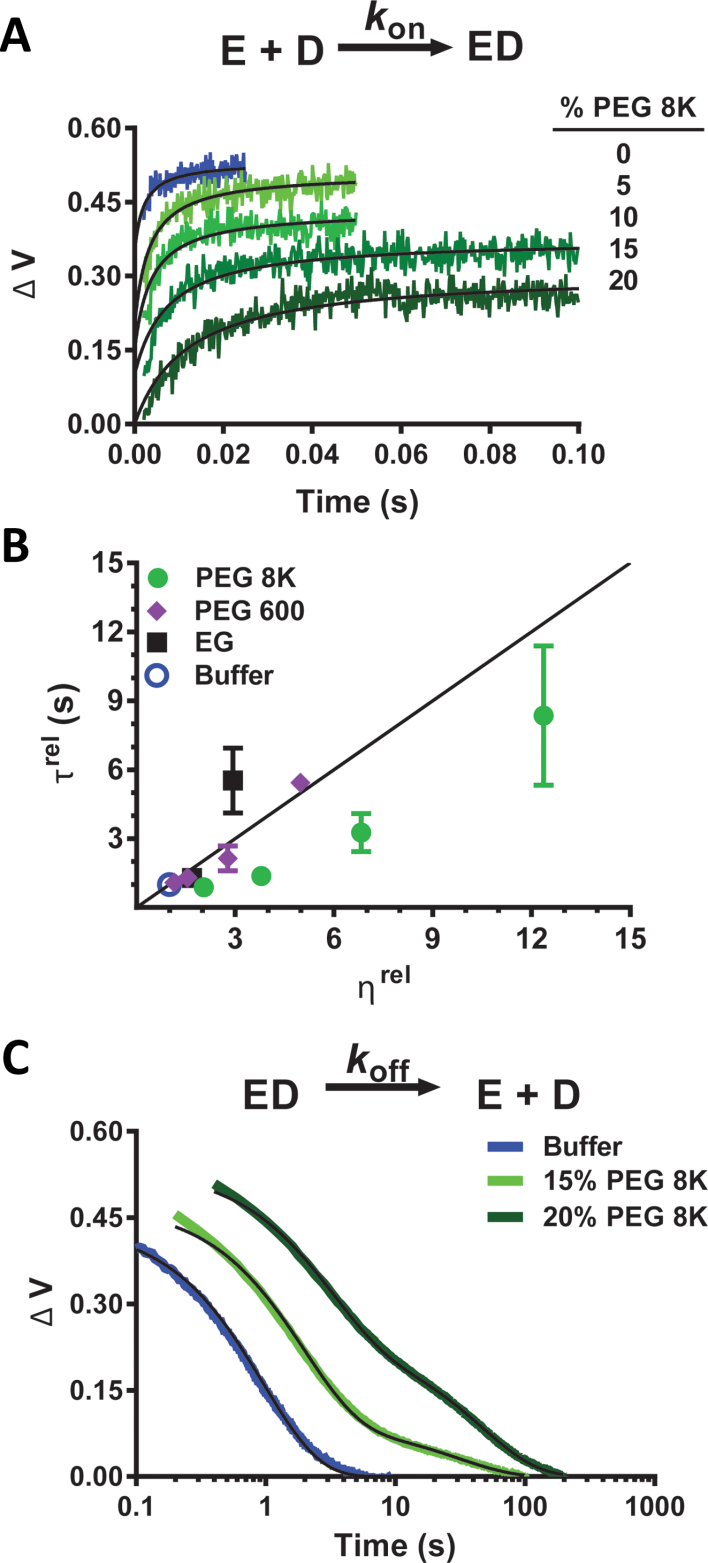
Influence of PEG 8K on association kinetics of hUNG from specific DNA (D^S^) determined by stopped-flow 2-aminopurine fluorescence measurements at 20°C. (**a**) Kinetic traces for the second-order association of hUNG (200 nM) with D^S^ (200 nM) in dilute buffer (blue) and in the presence of 5–20% PEG 8K. Traces are displaced along the y-axis for clarity. The second-order rate constants (*k*_on_) are reported in Supplementary Table S5. (**b**) Effect of relative viscosity (*η*^rel^ = *η*^crowder^/*η*^buffer^) on the relative association times (*τ*_rel_ = *τ*^crowder^/*τ*^buffer^, where *τ* = 1/*k*_on_) for hUNG and specific DNA (D^S^). The data points correspond to dilute buffer (blue) and increasing concentrations of EG (black), PEG 600 (purple) and PEG 8K (green). Relative viscosities of these cosolutes were obtained from (29). The theoretical line shows the expected dependence of the association times based solely on the increases in the relative viscosity of the solutions as expected from Stokes–Einstein behavior. (**c**) Effect of PEG 8K on the dissociation rate of hUNG from a specific site (D^S^). Kinetic traces are shown in a semi-log format. The dissociation kinetics follow a single exponential in buffer alone (blue), and a double exponential in the presence of 15% (light green) and 20% PEG 8K (dark green). The second slower exponential that appears in the presence of PEG 8K is deemed a fluorescence artifact as detailed in the Supplemental Methods. The kinetic parameters are reported in Supplementary Table S6.

In order to determine the origin of the minimal effect of crowding agents on DNA binding, we first investigated how the presence 20% PEG 8K alters the association rate (*k*_on_) of hUNG using a specific substrate analog by stopped-flow fluorescence methods (Figure 5a). These experiments used a 19mer duplex (D^S^) that contained a fluorescent 2-aminopurine (2-AP) base adjacent to a 2′-fluorinated deoxyuridine nucleotide (U^β^). The U^β^ nucleotide is resistant to glycosidic bond cleavage during the time frame of the measurements (46). Upon binding of hUNG, the uracil base of U^β^ is flipped into the active site, unstacking the 2-AP base and leading to an increase in its fluorescence intensity at 370 nm. Association rates were determined using second-order irreversible binding conditions in which equal molar amounts of hUNG and DNA (200 nM) were rapidly mixed in the presence of increasing amounts of PEG 8K (0–20%) and the kinetic traces were fit to Equation (3) (see ‘Materials and Methods’ section). As shown in Figure 5a and reported in Supplementary Table S6, *k*_on_ decreased markedly from 2.7 × 10^9^ to 3 × 10^8^ M^−1^ s^−1^ as the PEG 8K concentration was increased from 0 to 20%. The irreversibility of the association reactions was substantiated by the similar *k*_on_ values obtained using 100 nM concentrations of enzyme and DNA (*k*_on_ = 3.5 ± 0.7 × 10^9^ M^−1^ s^−1^ for dilute buffer; *k*_on_ = 2.9 ± 0.5 × 10^8^ M^−1^ s^−1^ for 20% PEG 8K). The association rate measured in dilute buffer approaches the fastest known rate constants for protein–DNA association (47) and requires that the steps subsequent to association leading to the 2-AP fluorescence increase are extremely rapid (i.e. base flipping and DNA/enzyme conformational changes) (5,46,48–50). Thus, binding of hUNG to this DNA in the absence of crowding is essentially encounter controlled.

We were curious as to whether the association rate attenuations observed with various concentrations of PEG 8K could be explained solely by the increases in relative viscosity (*η*^rel^) as the PEG 8K concentration was increased. To address this question we plotted the relative association times at each concentration of PEG 8K (*τ*^rel^ = *τ*^crowd^/*τ*^buffer^) against the relative viscosities (*η*^rel^) (29). We also performed the same plots using 20–40% EG and 5–30% PEG 600 to ascertain whether polymer size played a role (Figure 5b). These plots were compared with the expected linear correlation between *τ*^rel^ and *η*^rel^ predicted from the Stokes–Einstein (SE) equation (i.e. *τ*^rel^ = *η*^rel^, black line, Figure 5b). For EG (black squares), *τ*^rel^ increased in a steep parabolic fashion that deviated positively from the SE line at concentrations higher than 20% (positive deviations indicated slower association than expected from viscosity alone). This suggested inhibitory interactions of EG with hUNG when its concentration exceeds 20% (Figure 5b), which is consistent with what was observed in the steady-state kinetic measurements. In contrast, *τ*^rel^ for increasing concentrations of PEG 600 (purple circles), showed no significant deviation from SE behavior, indicating ideal viscogen behavior for this moderately small PEG polymer. Finally, *τ*^rel^ for increasing concentrations of PEG 8K (green diamonds) traced a concave curve that deviated negatively from the SE line (faster association than would be expected from viscosity alone).

### Crowding effects on the dissociation rate from a specific site (D^s^)

The effects of 5–20% PEG 8K on the dissociation rate from a specific site were determined by following the decrease in 2-AP fluorescence that accompanies hUNG dissociation from D^S^ (Figure 5c). In these experiments, an excess amount of DNA containing a high affinity abasic site (ϕDNA) was rapidly mixed with a pre-incubated solution of hUNG and D^S^ to ensure that all dissociated enzyme molecules were rapidly and irreversibly trapped (two ϕDNA concentrations were used to confirm this requirement). Although the dissociation rate in buffer alone was well fit to a single exponential decay with a rate constant *k*_off_ = 1.01 ± 0.05 s^−1^ (Figure 5c, Supplementary Table S7), the presence of PEG 8K resulted in double-exponential decays of the fluorescence (Figure 5c). Both the rate and amplitude of the first rapid phase decreased with increasing PEG 8K concentrations (Supplementary Table S7). At the final concentration of 20% PEG 8K, the rate of the fast transient (*k*_off_^PEG^ = 0.35 s^−1^) was ∼3-fold less than the buffer only value and the amplitude was reduced by 40%. The rate constant for the slower phase (*k*_slow_ = 0.024 s^−1^) was 50-fold smaller than the value for *k*_off_ in buffer only, and the amplitude of the slower kinetic phase increased with PEG 8K concentration to a final value that was 44% of the total fluorescence change (Figure 5c, Supplementary Table S7). In the Supplemental Discussion we discuss why the slow kinetic transient can only reasonably be attributed to a fluorescence artifact unrelated to the dissociation rate of hUNG from D^S^. Accordingly, we only consider the *k*_off_^PEG^ = 0.35 s^−1^ in our analyses of the effects of 20% PEG 8K on DNA binding and translocation (see below).

## DISCUSSION

**Figure 6.**
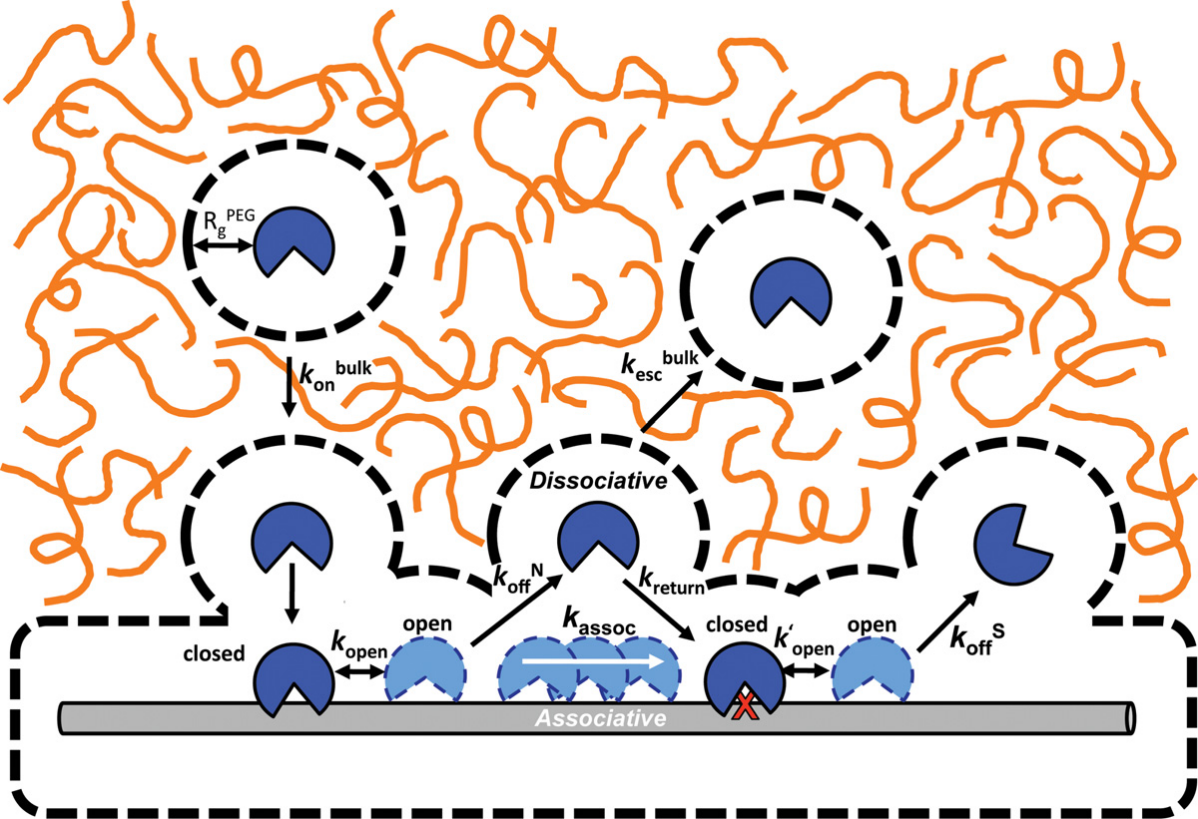
General schematic of how the introduction of molecular crowders (orange lines) can influence individual steps in the DNA glycosylase damage search pathway. These steps including the rate of diffusion to the DNA chain (*k*_on_), the lifetime of nonspecific (1/*k*_off_^N^) and specific (1/*k*_off_^S^) DNA complexes, the probability of associative and dissociative transfers between damage sites, and changes in the rate of product release (rate-limiting for *k*_cat_). Dashed lines represent the sizes of the depletion layers surrounding the protein and DNA where the PEG 8K polymer, but not solvent, is excluded. For large DNA molecules, association is limited by translation of the protein through the crowded solution (*k*_on_^bulk^) until their depletion layers overlap and association proceeds within a low viscosity environment. Also depicted are highly dynamic closed-to-open conformational changes in hUNG and hOGG1 that accompany nonspecific DNA binding (42,50); only the open state is viewed competent for translocation (50). Release of each enzyme from the product requires an even larger closed-to-open transition that has been shown to be at least partly rate-limiting for turnover of hUNG (*k′*_open_) (46). Image is drawn to scale using a DNA duplex of 2 nm as a scale reference. The depletion layer size in the dilute regime for PEG 8K is shown (equivalent to *R*_g_^PEG^, see Supplemental Information).

In this study, we have developed an *in vitro* model that approximates some aspects of the crowded environment of the cell nucleus in order to explore how molecular crowding affects the DNA damage search and repair pathway of two human DNA glycosylases. The extensive results can be largely explained by considering both the macroscopic and microscopic effects of large polymers on solution viscosity and excluded volume. These effects are depicted in Figure 6 for the search coordinate of hUNG, which will be the focus of the following discussion given that the bulk of the experiments were performed on this enzyme. While we have not explicitly determined the effects for each step of the search coordinate of hOGG1, the generality of the conclusions are inferred based on the similarities of the effects of molecular crowding on facilitated diffusion of both enzymes. Below, we interpret the results based on the sequential steps shown in Figure 6 (left to right), beginning with bulk diffusion of the enzyme to the DNA chain and ending with its departure to bulk solution after encountering the specific site. For hUNG, the forward steps leading to excision of the uracil base are much faster than dissociation from the specific site (46); these steps are not shown in Figure 6 for clarity.

### Translational diffusion from bulk solution to DNA

Addition of PEG 8K resulted in the attenuation of the bimolecular encounter rates compared to buffer (*k*_on_, *k*_cat_/*K*_m_), but to a lesser extent than expected from viscosity changes alone (Figure 5b). Association of hUNG with a large DNA chain requires long-range translation through solution, which will be slowed by macroscopic viscosity according to the Stokes-Einstein (SE) relation *D*_t_ = *k*_B_*T*/6*πηR*, where *η* is the solution viscosity and *R* is the hydrodynamic radius of the diffusing species. However, the diffusion limit imposed by viscosity can be substantially reduced when the cosolute is a crowder that occupies a solution volume larger than the diffusing species (8). This effect arises from the preferential interaction of water with the protein (and DNA) resulting in a hydration layer in which the crowder is excluded (termed the ‘depletion layer’). For PEG and other random coil polymers, the depletion layer scales as a function of the radius of gyration of the polymer (*R*_g_) (35). (The basis for the depletion layer sizes that we use in our qualitative analysis is found in Supplemental Methods.) Accordingly, the depletion layer for PEG 8K (∼4.1 nm) is calculated as ∼4.5-fold larger than PEG 600 (∼0.9 nm) (29,51). The comparatively large depletion layer for PEG 8K indicates that when the centers of mass of hUNG and the DNA approach within ∼11 nm of each other (see Supplemental Methods), binding will efficiently ensue, partially ameliorating the effects of bulk viscosity on translational diffusion. The significantly thinner depletion layer of PEG 600 (Figure 5b) accounts for its adherence to SE behavior because hUNG and the DNA must come in much closer proximity before the low viscosity depletion zone is formed. We conclude that the kinetic effects of PEG 600 and PEG 8K on bimolecular encounter are accounted for by viscosity alone (PEG 600), or antagonistic viscosity and caging effects derived from the large depletion layer of PEG 8K.

### Associative transfer steps

A major finding was that crowding agents dramatically increased the probability and mean distance for associative transfers on the DNA chain (Figure 2b). We propose that this effect derives from two distinct properties (i) the fact that associative transfers occur with the enzyme still located within the ion cloud of the DNA (<2 nm) (30), and therefore, also within the low viscosity depletion layer, and (ii) the caging effect of crowders that would tend to reflect the enzyme back to the DNA chain during dissociation attempts. The increased time spent in associative transfers would allow for more comprehensive local damage surveillance, but at the expense of moving frequently to other DNA chains. This comprises the basis for why removal of uracils embedded in a population of long 1U^90^ substrate molecules is slowed significantly in the presence of crowding, but smaller DNA substrates do not show the same behavior (Figure 4e, Supplementary Table S5). Such a local search mechanism is highly appropriate for the human cell nucleus because glycosylases are typically abundant enzymes (>100 000 copies per nucleus) (7), and each enzyme only needs to scan less than 20 000 bp of DNA on average. Thus, there is no great need for individual enzyme molecules to move through a large volume to affect repair.

### Dissociative transfer steps

In contrast with the substantial enhancement of the associative transfer pathway in the presence of 20% PEG 8K, only a small effect was observed for the dissociative pathway (*P*_diss_). This result may be rationalized using the kinetic definition of *P*_diss_ = [*k*_off_/(*k*_off_ + *k*_assoc_)][(*k*_return_/(*k*_return_ + *k*_bulk_)] (30). The first term describes the likelihood that an enzyme molecule will dissociate from the DNA as opposed to proceeding through an associative step (the inverse of the kinetic definition of *P*_assoc_). For the same reasons discussed above for associative transfers, this first term would be reduced in the presence of crowders. The second term describes the probability that a dissociated enzyme molecule will return to the DNA chain (*k*_return_) or become lost to bulk solution (*k*_bulk_). Since the low viscosity depletion layer extends to ∼10 nm we expect that the majority of the dissociated enzyme molecules will return to the DNA chain, and we also surmise that escape to bulk will be impeded in heavily crowded and viscous solutions. Thus, the second term of this expression is expected to increase. We propose that opposing changes in the first and second terms nearly cancel, resulting in the overall small change in *P*_diss_ that is observed.

### Departure to bulk solution

The presence of PEG 8K resulted in similar decreases (3- to 4-fold) in the dissociation rates from a specific site (*k*_off_^PEG^) and product sites derived from the 6U^11^ and 1U^30^ substrates (in this comparison we use *k*_cat_ as a surrogate for the dissociation rate from the product site) (Figure 5c, Supplementary Table S7). These common decreases are likely the result of increased solution viscosity and/or inefficient escape of the enzyme from the depletion layer around the DNA chain due to collision with the polymer cage. Finally, the 2–3 fold increases in *K*_D_^NS^, *K*_D_^S^ and the substrate *K*_m_ values in the presence of 20% PEG 8K are accounted for by the compensatory changes in the association rate (a decrease of ∼10-fold, Supplementary Table S6) and dissociation rate (a decrease of ∼3-fold, Supplementary Table S7).

### Damage search and repair in the cell nucleus

This study moves us one step closer to understanding how environmental factors within the human cell nucleus could modulate the activity of DNA repair enzymes. While further *in vitro* experiments will be designed to even more closely mimic the nuclear environment, our clearest understanding of DNA search and repair will ultimately require measurements in human cells. Nevertheless, the systematically studied *in vitro* behaviors will be essential for useful interpretation of the behaviors observed in the context of a complex nuclear milieu.

## SUPPLEMENTARY DATA

Supplementary Data are available at NAR Online.

SUPPLEMENTARY DATA
