# Supplementary material for: Molecular crowding enhances facilitated diffusion of two human DNA glycosylases
Source: Nucleic Acids Res. 2015 Apr 6;43(8):4087–97. doi: 10.1093/nar/gkv301 (PMC4417188; doi:10.1093/nar/gkv301)
Supplement: SUPPLEMENTARY DATA [file supp_43_8_4087__index.html]

Molecular crowding enhances facilitated diffusion of two human DNA glycosylases — Molecular crowding enhances facilitated diffusion of two human DNA glycosylases — SUPPLEMENTARY DATA 

# Molecular crowding enhances facilitated diffusion of two human DNA glycosylases

## SUPPLEMENTARY DATA

**Files in this Data Supplement:**

- SUPPLEMENTARY DATA
