# Supplementary material for: Molecular crowding enhances facilitated diffusion of two human DNA glycosylases
Source: Nucleic Acids Res. 2015 Apr 6;43(8):4087–97. doi: 10.1093/nar/gkv301 (PMC4417188; doi:10.1093/nar/gkv301)
Supplement: SUPPLEMENTARY DATA [file supp_gkv301_nar-00047-f-2015-File008.pdf]

Supplemental Tables and Figures for
Molecular Crowding Enhances Facilitated Diffusion of Two Human DNA Glycosylases

Shannen L. Cravens, Joseph D. Schonhoft, Meng M. Rowland, Alyssa A. Rodriguez, Breeana G. Anderson and James T. Stivers

Methods

***DNA Sequences*.** All DNA's are double stranded with the exception of the hairpin $6U^{11}$. The complimentary strand sequences are implied based on normal Watson-Crick pairing but not shown below. All uracils and tetrahydrofuran residues are paired with A on the opposite strand, except where indicated, and 2-aminopurine is paired with T. 8-oxoG is always paired with C on the complimentary strand.

hUNG

D^N: 5'-FAM-AGG CGC ATA GTC GCA-3'

D^S: 5′-GCG GCC AA PU^βA AAA AGC GC-3′ (U^β/G mismatch; P - 2-aminopurine)

ϕ***DNA****:* 5′-GCG GCC AAA ϕ AA AAA GCG C-3′ (ϕ - tetrahydrofuran; *ϕ-A pair*)

Substrates for Steady-State Kinetics

$6U^{11}$ (hairpin): 5'-FAM-GCA **UU**A AGA AGA AG-$(PEG)_6$-C**UU** C**UU** AAT TGC-BHQ-3'

$1U^{90}$ (90mer): 5′ - GTT ATC CGC TCA CAA TTC CAC ACA ATG CTG AGG AAT CGA **U**AG CTA AGT AGG ATG TTA GCT ATC GAT TCA TCC TCA GCA CAG TGT CGA GCC - 3′

$1U^{30}$ (30mer): 5′ - CGT AGC CAC TGC AAP **U**AA ACA GAG CAT AGG - 3′

Two-Site Substrates

$S10^U$ (90mer): 5′ - GGT ATC CGCT AGT CAC AAT TCC ACA CAATGC TGA GGA ATC GA **U** AG CTA AT CGA **U** AGC TAA GCT GAG GCATAC AGG ATC AAT TGT CGA GCC-3′

S20^U (90mer): 5′-GGT ATC CGC TCA CAA TTC CAC ACA ATG CTG AGG AAT CGA **U** AG CTA AGT AGG ATG AAT CGA **U** AG CTA AGC TGA GGC ATA CAG TGT CGA GCC - 3′

S55^U (125mer): 5'-GGT ATC CGC TCA CAA TTC CAC ACA ATG CTG AGG AAT CGA **U** AGC TAA GTG AAT CTC TCA CGT CAC ATC GTC CGC ACT AGC ACA TGG AAT GAA TCG A **U** AGC TAA GCT GAG GCA TAC AGT GTC GAG CC - 3'

hOGG1

S0^{oG} (31mer): 5′-ATG CTG AGG AAT TTC $^{\mathbf{o}}$**G**CT CCT TGT AGG ATG A-3′

S20^{oG} (90mer): 5´-GGT ATC CGC TCA CAA TTC CAC ACA ATG CTG AGG AAT TTC $^{\mathbf{o}}$**G**CT CCT TGT AG G ATG A AT TTC $^{\mathbf{o}}$**G**CT CCT TGC TGA GGC ATA CAG TGT CGA GCC-3´

hUNG Equilibrium DNA Binding Measurements. Binding of hUNG to a nonspecific 5' fluorescein-labeled 15mer DNA duplex (D^N) was measured by fluorescence anisotropy using a SPEX Fluoromax-3 spectrofluorometer at 20 °C (excitation wavelength of 494 nm, emission wavelength of 518 nm). All anisotropy values were corrected for the spectral G factors. Experiments were performed in a back-titration mode by mixing a solution of concentrated hUNG containing 100 nM labeled DNA with increasing volumes of a solution containing 100 nM labeled DNA only. Experiments were performed with the above buffer in the absence and presence of 20% (w/v) PEG 8K. After each addition, the solution was allowed to equilibrate for 4 min inside the fluorometer and three anisotropy measurements were averaged. All data were fitted to eq 2 using the software Prism, where A_0 and A_f are the minimal and maximal anisotropy values, respectively.

$$\mathrm{A} = -\left\{\frac{(\mathrm{A}_0 - \mathrm{A}_\mathrm{f})}{2} \times [\mathrm{DNA}]_{\mathrm{tot}}\right\} \times \left(\mathrm{b} - \sqrt{\mathrm{b}^2 - 4[\mathrm{hUNG}]_{\mathrm{tot}}[\mathrm{DNA}]_{\mathrm{tot}}}\right) + \mathrm{A}_0 \quad (2)$$

$$\mathrm{b} = (K_\mathrm{D}) + [\mathrm{hUNG}]_{\mathrm{tot}} + [\mathrm{DNA}]_{\mathrm{tot}}$$

hOGG1 Expression and Purification. E. coli BL21(DE3) Trigger Factor cells were transformed with pET30a(+) (Novagen) plasmid encoding wild type hOGG1 (residues 1-345) with an N-terminal His_6 tag. Expressions used 2 liters of LB containing 40 μg/mL kanamycin and 34 μg/mL chloramphenicol. Culture broth

was inoculated with the transformed cells and then incubated at 37 °C until OD_{600} = 0.6. hOGG1 expression was induced by the addition of 0.5 mM IPTG and the culture was further incubated at 16 °C overnight. Cells were then harvested by centrifugation and frozen at -80 °C overnight. Cells were resuspended in lysis buffer (50 mM Tris pH 7.5, 300 mM NaCl, 5% glycerol, 0.1% Triton-X 100, 1 mM EDTA, 1 mM DTT, 0.25 mg/mL lysozyme). The supernatant was clarified by centrifugation at 40,000g for 40 min at 4 °C) and then batch bound to 3 mL of Ni-NTA agarose (Qiagen) by rocking the lysate (supplemented with 5 mM imidazole) and column resin for 1 hour at 4 °C. The resin was pelleted by centrifugation at 4,000x rpm for 10 min and the supernatant was discarded. The column was rocked for 10 min at 4 °C with buffer A (20 mM Tris-Cl, pH 7.5, 1 mM DTT, 10% glycerol) containing 5 mM imidazole to remove any nonspecifically bound protein. After pelleting the resin and discarding the supernatant, the resin was rocked for 10 min at 4 °C with buffer A containing 500 mM imidazole to remove all specifically bound protein. The resin was pelleted and the supernatant was diluted 3-fold and poured directly onto a Mono-S cation exchange column (GE Healthcare) preequlibrated in buffer A. hOGG1 was then purified by gradient elution with buffer A containing 1 M NaCl. Fractions containing hOGG1 were pooled and diluted to final buffer conditions of 20 mM Tris-Cl pH 7.5, 300 mM NaCl, 1 mM DTT, 20% glycerol and stored at -80 °C. The N-terminal His_6 tag was not removed given previous reporting in the literature that removal of the His_6 tag resulted in no difference in single-turnover rate constants (1). Purification yielded protein that was 30−50% active, determined by single-turnover active site titrations. All hOGG1 protein concentrations reported correspond to active enzyme concentrations.

Measurement of hOGG1 Reaction Rates. Stock solutions of hOGG1 must be diluted carefully to obtain reproducible kinetic measurements. Empirically we have found that the following procedure provides reproducible results. hOGG1 stock (6 µM) can be stored at -80 ^{o}C for at least several months without any observed decrease in activity. 2 µL of a 6 µM hOGG1 stock in storage buffer (20mM Tris-HCl pH 7.5, 300mM NaCl, 20% glycerol) was diluted to 20 µL using buffer B (20 mM Tris-HCl pH7.5, 500 mM NaCl, 20% glycerol, and 150 µg/mL BSA). The resulting enzyme solution was incubated at room temperature for 5 min before 12-fold dilution in buffer B to give a final concentration of 50 nM. One-microliter of this diluted enzyme solution was used to initiate each 50 µL reaction (final [hOGG1] = 1 nM). hOGG1 (1 nM) was reacted with 20 nM ^{32}P-labeled substrate ($S0^{oG}$) in buffer A at 37 ˚C. As needed, the buffer contained 20% (w/v) PEG 8K or 5% (w/v) of Hemoglobin in a total volume of 50 µl. At 2, 4, 6, 8, and 10 min, 6 µL portions of the reaction mixtures were

quenched with 20 μL of formamide loading buffer and heated for 10 min at 95 ˚C to generate a 15mer cleavage product that could be visualized after electrophoretic separation using a 10% polyacrylamide gel containing urea. The fractional extent of reaction at each time was quantified by phosphorimaging of the gels and the reaction rates were determined from the linear slopes of plots of product concentration against time using Prism and averaging over three trials.

Calculation of Depletion Layer Sizes. In the dilute regime, polymers can be considered as non-interactive random coils with a defined radius of gyration (R_g^{PEG}) that is dependent on the molecular weight (M_w) of the polymer ($R_g^{PEG} = 0.0215 \cdot M_w^{0.583}$) (2, 3). The depletion layer surrounding the protein and DNA in this regime is considered equivalent to R_g^{PEG} (4, 5). As the concentration of polymer is increased beyond a defined crossover threshold (c*) (5), the solution enters the semi-dilute regime, where polymer molecules overlap and the protein and DNA are embedded in a noncovalent polymeric network with a certain average mesh size (ξ) that continues to decrease as the polymer concentration increases. The depletion layer around the protein and DNA also decreases with the mesh size, which is a complex function of R_g^{PEG} (5). For simplicity, we chose to calculate the relative sizes of the depletion layers generated by semi-dilute solutions of PEG using the simple approximations for the dilute regime (4.1 nm for PEG 8K and 0.9 nm for PEG 600). Using this model, the depletion layers surrounding the protein and DNA would overlap when centers of mass of both species reach a distance equal to the combined radii of the two depletion layers (2 x R_g^{PEG}) plus the sum of the Stokes radii for the protein and DNA ($R_{Stokes}^{hUNG} + R^{DNA}$, where R_{Stokes}^{hUNG} = 2.3 nm (6) and R^{DNA} ~ 1 nm for B-form DNA). While it is true that the depletion layer would begin to decrease in the semi-dilute regime as concentration of PEG was increased, the depletion layer for PEG 8K would still be larger than PEG 600 in this regime. Our qualitative analysis is not dependent on whether the depletion layer sizes for PEG solutions are precisely known.

Supplemental Discussion

Effect of Hemoglobin on Translocation of hUNG. Site transfer experiments conducted with hUNG were complicated by a dramatic reduction in the reaction rate of uracil excision in the presence of in 5% (w/v) hemoglobin (**Supplementary Figure S3**). Despite this non-ideality, the data revealed an apparent directional bias for transfer of hUNG along the DNA, which can be discerned by the enhanced production of the double

cleavage product band C relative to A (see analysis below and **Supplementary Figure S4a**). We ensured this result was not due to hemoglobin altering the electrophoretic migration of the DNA through the gel by control experiments that verified equivalent amounts of intact, single, and double cleaved DNA fragments were observed on a gel when loaded in the presence and absence of hemoglobin.

Analysis of the reaction velocities for each product band revealed a burst phase followed by a slower linear rate (**Supplementary Figure S4b**). The amplitude of the burst phase was independent of [hUNG] in the range 100 to 500 pM hUNG, indicating that it does not arise from slow ES turnover in the presence of hemoglobin. The burst phase likely results from slow onset inhibition of hUNG upon its addition to the solution containing hemoglobin, resulting in a steady-state level of free E that is maintained over the remaining course of the reaction. In contrast, the linear steady-state rates were about 10 times slower when 100 pM hUNG was used as compared to 500 pM.

We analyzed the site excision data in the presence of hemoglobin using the model shown in **Scheme 1**.

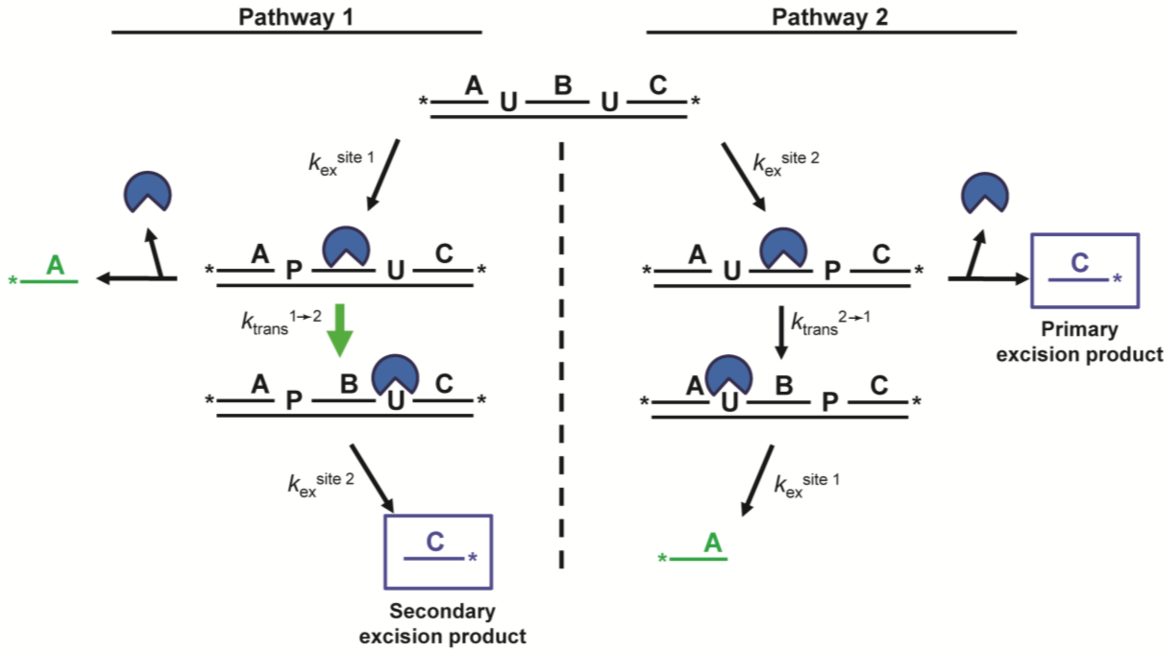

Scheme 1. Site transfer mechanism consistent with hUNG behavior in the presence of hemoglobin. k_{ex} is the initial rate of primary excision events at either uracil site, which may then result in dissociation of the enzyme before encounter of the second site, or alternatively, transfer to that site (k_{trans}). The biased directionality of site transfers is indicated by arrows. Thick, green arrows indicate steps favored in the presence of hemoglobin and the color-coding of DNA fragments coincides with **Figure S4**. The major C product fragment is boxed for clarity.

Although we have previously established that it is not possible to distinguish whether apparent directional bias in site transfer arises from differences in the excision rates at each site (k_{ex}) or true directionality to site transfer (7), we believe the current results are most reasonably interpreted in terms of transfer effects. Our reasoning is that it seems highly unlikely that hemoglobin would give rise to different effects on site excision because both sites are identical. Using the reasonable assumption that k_{ex} is the same for both sites, then the high levels of fragment C in Figure S4a (derived from pathways 1 and 2 in **Scheme 1**) may be explained using eq S1, where the fragment concentrations are obtained by extrapolation to zero time (**Fig. S4c**). This equation

$$P_{trans}^{1\rightarrow 2} = \frac{[A]_0 - [BC]_0}{[A]_0} \qquad \text{(S1)}$$

isolates how much fragment C was generated via pathway 1 by consumption of fragment BC after translocation of the enzyme from site 1→2 (**Scheme 1**). Although fragment A can also be made by secondary excision via pathway 2, this cannot be a significant occurrence because fragment AB is always present at a much higher level than BC. The observed $P_{trans}^{1\rightarrow 2}$ value of 0.56 ± 0.05 is higher than P_{trans} determined in buffer (0.33 ± 0.09) and was unchanged in the presence of 20 mM uracil trap (P_{trans} = 0.64 ± 0.02), suggesting that the biased transfers are associative in nature (**Figure S4c**). Although these data can be interpreted in terms of biased transfer in the presence of hemoglobin, we concede that the data could arise from unknown complications and are therefore unable to definitely conclude that a protein crowder increases hUNG translocation.

Analysis of the Biphasic Dissociation Kinetics from the Specific site (D^S). We conducted a number of experimental and theoretical evaluations to determine if the slower kinetic transient for dissociation of D^s in the presence of PEG 8K reflected an important aspect of the dissociation reaction, or alternatively, was an artifactual 2-AP fluorescence change resulting from the presence of the PEG polymer. We excluded the possibility that 20% PEG 8K produced detectable 2-AP or hUNG tryptophan fluorescence changes by mixing the enzyme or D^S DNA alone with the ΦDNA trap in the stopped flow device and following the fluorescence emission at 370 nm over time. Although this control did not reveal any detectable fluorescence changes, as observed when enzyme and D^S were mixed, the 50-fold slower kinetic transient in the traces shown in **Figure 5c** is not compatible with many other measurements contained in this study. One inconsistency is that an

exceedingly slow transient in the presence of 20% PEG 8K should reduce the net rate of substrate dissociation and also reduce K_m, yet only a 4-fold decrease in K_m was observed for a substrate of similar length as D^S ($1U^{30}$). Also, we have previously found that the kinetic process and conformational changes for UNG dissociation from a substrate analogue site and a product site are very similar, which suggests that k_{cat} is a good surrogate for the product dissociation rate (8-11). Accordingly, the ~4-fold decreases in k_{cat} that were observed for the short substrates $1U^{30}$ and $6U^{11}$, more closely resemble the 3-fold reduction in the fast kinetic phase for dissociation of D^S in **Figure 5c,** and not the 50-fold slower kinetic phase. Finally, if the dissociation rate of D^S is assumed to be equivalent to the fast kinetic phase, the calculated $K_D{}^S$ in the presence of 20% PEG 8K is $k_{off}{}^{PEG}/k_{on}{}^{PEG} = 1.2 \pm 0.5 \times 10^{-9}$ M, which is about 3-fold greater than $K_D{}^S$ in the absence of PEG 8K (**Supplementary Table S6**). This effect of PEG 8K on specific binding is similar to the ~2-fold increase in K_D observed for non-specific binding and the modest ~3-fold increases in the substrate K_m values. We thus conclude that the significantly slower phase observed in the presence of PEG 8K does not reflect on the dissociation rate of hUNG from a specific site on DNA.

Supplemental Table S1. Solution viscosities at 25 ˚C for varying amounts of EG, PEG 600, PEG 3350, and PEG 8K (literature values) (4).

**% mass (w/v)**	**$\eta^{crowd}/\eta^{buffer}$**			
	**EG**	**PEG 600**	**PEG 3350**	**PEG 8K**
**5**	1.12	1.15	1.57	2.04
**10**	1.32	1.55	2.54	3.79
**15**	1.48	2.11	4.12	6.83
**20**	1.68	2.77	6.60	12.37
**25**	1.88	3.69	--[a]	--
**30**	2.18	4.98	--	--
**40**	2.93	--	--	--

[a]Value could not be determined from literature source.

Supplemental Table S2. Sodium ion activity in the presence and absence of 20% PEG 8K

	**Na^+ Activity ($mV \cdot M^{-1}$)**
Buffer	49 ± 1
20% PEG 8K	41 ± 3

Supplemental Table S3. P_{trans}, P_{assoc}, and P_{diss} for hUNG in the presence and absence of 20% PEG 8K

**Site Spacing (bp)**	**P_{trans}**		**P_{assoc}[a]**		**P_{diss}[b]**	
	**Buffer**	**20% PEG 8K**	**Buffer**	**20% PEG 8K**	**Buffer**	**20% PEG 8K**
5	0.60 ± 0.04	0.92 ± 0.06	0.37 ± 0.06	0.61 ± 0.09	0.23 ± 0.07	0.3 ± 0.1
10	0.45 ± 0.04	0.91 ± 0.06	0.03 ± 0.04	0.44 ± 0.08	0.46 ± 0.04	0.5 ± 0.1
20	0.33 ± 0.09	0.71 ± 0.06	0.02 ± 0.07	0.26 ± 0.03	0.33 ± 0.09	0.45 ± 0.07
55	0.21 ± 0.06	0.50 ± 0.08	-0.08 ± 0.07	0.14 ± 0.06	0.21 ± 0.06	0.4 ± 0.1

[a] P_{assoc} was determined by performing the site transfer experiment in the presence of 10 mM uracil and was independent of uracil concentration in the range 10 to 20 mM.

[b] Calculated from $P_{diss} = P_{trans} - P_{assoc}$

Supplemental Table S4. Effect of 20% PEG 8K on P_{trans}, P_{assoc}, and P_{diss} for hOGG1 (**$S20^{oG}$**).

**Site Spacing (bp)**	**P_{trans}**		**P_{assoc}[a]**		**P_{diss}[b]**	
	**Buffer[c]**	**PEG8K**	**Buffer[c]**	**PEG 8K**	**Buffer[c]**	**PEG 8K**
20	0.34 ± 0.09	0.50 ± 0.03	0.14 ± 0.05	0.37 ± 0.03	0.20 ± 0.11	0.13 ± 0.02

[a] P_{assoc} was determined by performing the site transfer experiment in the presence of 3 mM trap.

[b] Calculated from $P_{diss} = P_{trans} - P_{assoc}$

[c]Data has been previously published and is presented here for comparison (12).

Supplemental Table S5. Effects of EG and PEG 8K on the steady-state kinetic parameters of hUNG with short and long DNA substrates.[a]

**DNA Substrate**		k_{cat} (s^{-1})	K_m (M)	k_{cat}/K_m (M^{-1} s^{-1})
	Buffer	3.0 ± 0.1	11 ± 1 x 10^{-9}	2.8 ± 0.3 x 10^{8}
	20% EG	1.6 ± 0.1	23 ± 6 x 10^{-9}	7 ± 2 x 10^{7}
**6U**[11]	40% EG	1.4 ± 0.2	70 ± 30 x 10^{-9}	2 ± 1 x 10^{7}
(11mer)[b]	5% PEG 8K	1.7 ± 0.2	4 ± 1 x 10^{-9}	5 ± 2 x 10^{8}
	10% PEG 8K	1.39 ± 0.05	5.2 ± 0.7 x 10^{-9}	3.2 ± 0.5 x 10^{8}
	20% PEG 8K	0.76 ± 0.06	3 ± 1 x 10^{-9}	1.9 ± 0.8 x 10^{8}
**1U**[30]	Buffer[c]	1.70 ± 0.04	120 ± 10 x 10^{-9}	1.4 ± 0.2 x 10^{7}
(30mer)	20% PEG 8K	0.39 ± 0.06	50 ± 20 x 10^{-9}	8 ± 3 x 10^{6}
**1U**[90]	Buffer[c]	4.7 ± 0.3 [c]	13 ± 3 x 10^{-9} [c]	4 ± 1 x 10^{8} [c]
(90mer)	20% PEG 8K	0.20 ± 0.02	4 ± 2 x 10^{-9}	5 ± 3 x 10^{7}

[a]The substrates used for the kinetic measurements have different sequences surrounding the uracil sites, which gives rise to modest differences in the kinetic parameters arising from sequence (13, 14). Thus, the fold changes in the kinetic parameters resulting from the addition of PEG are the most relevant parameters to compare.

[b]The kinetic parameters for this substrate reflect the excision of multiple uracils in a single encounter event that then result in a fluorescence increase due to hairpin destabilization. Thus, the rate units are oligonucleotide reacted per second rather than sites excised per unit time.

[c]Data has been previously published and is presented here for comparison (15).

Supplemental Table S6. Specific DNA association rates (k_{on}) for hUNG in the presence of a variety of molecular crowding agents compared to the predicted rate from the Stokes-Einstein relation (k_{on}^{SE}).

		5%		10%		15%		20%		30%		40%	
	$\boldsymbol{k_{on}}^{a}$	$\boldsymbol{k_{on}}$	$\boldsymbol{k_{on}}^{SE,b}$	$\boldsymbol{k_{on}}$	$\boldsymbol{k_{on}}^{SE}$	$\boldsymbol{k_{on}}$	$\boldsymbol{k_{on}}^{SE}$	$\boldsymbol{k_{on}}$	$\boldsymbol{k_{on}}^{SE}$	$\boldsymbol{k_{on}}$	$\boldsymbol{k_{on}}^{rel}$	$\boldsymbol{k_{on}}$	$\boldsymbol{k_{on}}^{SE}$
Buffer	27 ± 7	--[c]	--	--	--	--	--	--	--	--	--	--	--
EG	--	--	--	--	--	--	--	20 ± 1	18 ± 5	--	--	4.8 ± 0.4	11 ± 3
PEG 600	--	25 ± 8	23 ± 6	21 ± 1	17 ± 5	--	--	12.6 ± 0.4	10 ± 3	5.0 ± 0.4	7 ± 2	--	--
PEG 8K	--	31 ± 1	13 ± 3	20 ± 9	7 ± 2	8 ± 3	4 ± 1	3 ± 1	2.2 ± 0.6	--	--	--	--

[a]k_{on} has units of 10^8 $M^{-1}s^{-1}$.

[b]$k_{on}^{SE} = k_{on}^{buffer}/\eta^{rel}$

[c]Not determined.

Supplemental Table S7. Rate constants and amplitudes for slow and fast kinetic phases for the biphasic dissociation of hUNG from specific DNA in the presence of various amounts of PEG 8K.

	$k_{off}^{buffer\ a}$ (s^{-1})	k_{off}^{PEG} (s^{-1})	$k_{slow}^{\ b}$ (s^{-1})	$A^{PEG,\ c}$	$A^{slow,\ c}$	$K_D^{S\ d}$ (nM)
Buffer	1.01 ± 0.05	-- [e]	--	--	--	0.4 ± 0.1
5%	--	0.97 ± 0.05	0.048 ± 0.009	0.84	0.16	0.31 ± 0.02
10%	--	0.48 ± 0.01	0.022 ± 0.001	0.78	0.22	0.2 ± 0.1
15%	--	0.57 ± 0.02	0.035 ± 0.003	0.81	0.19	0.7 ± 0.3
20%	--	0.35 ± 0.02	0.024 ± 0.004	0.56	0.44	1.2 ± 0.5

[a] k_{off} is a single exponential in the absence of PEG 8K.

[b] k_{slow} is attributed to an artifact resulting from 2-AP fluorescence interference by PEG 8K (see Supplemental Methods).

[c] Fractional amplitudes of each kinetic phase.

[d] Calculated based on $K_D = k_{off}^{PEG}/k_{on}$.

[e] Not applicable

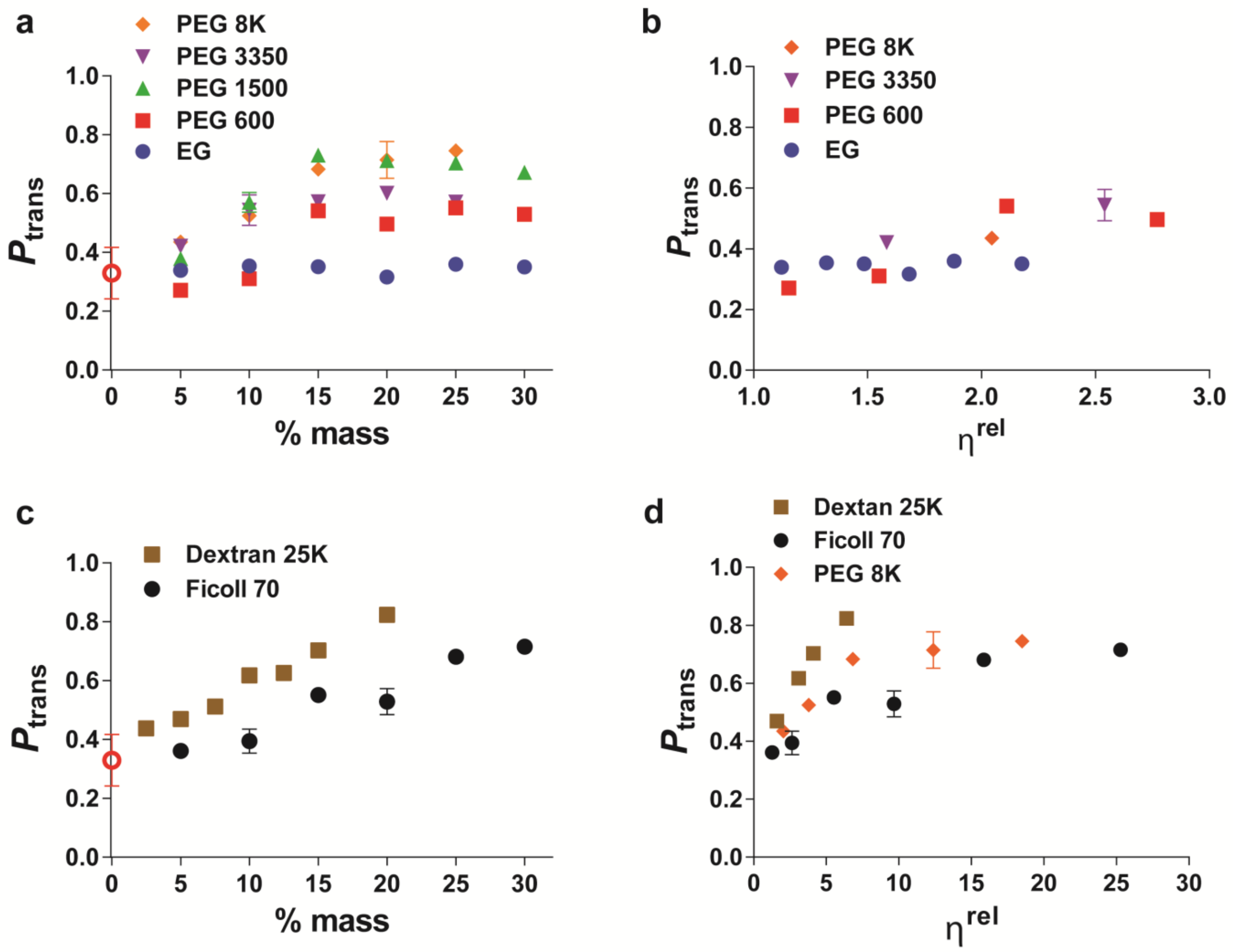

Supplementary Figure S1. Effect of molecular crowding agents on the site transfer probability (P_{trans}) of hUNG between uracil lesions spaced 20 bp apart. (a) Overall site transfer probabilities (P_{trans}) plotted as a function of % mass for ethylene glycol (EG) and other high MW PEG polymers. (b) Expansion of Figure 1c to highlight the minimal changes in P_{trans} at low relative solution viscosities ($\eta^{rel} = \eta^{crowd}/\eta^{buffer}$). (c) P_{trans} as a function of increasing amounts of Dextran 25K and Ficoll 70. (d) P_{trans} for Dextran 25K, Ficoll 70 (Figure S1c), and PEG 8K (Figure S1a) plotted as a function of relative solution viscosity. Relative viscosities for were taken from the literature ((16) for Dextran 25K and (17) for Ficoll 70). Only the data corresponding to solution conditions equivalent to those from the literature sources were used.

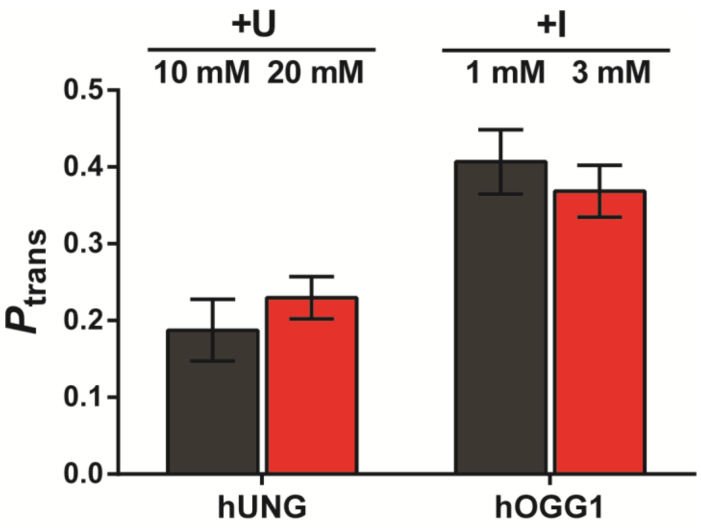

Supplementary Figure S2. The amount of associative transfers between lesions spaced 20 bp apart measured in the presence of PEG 8K are independent of the trap concentration for both hUNG and hOGG1. Both uracil and the hOGG1 inhibitor (I) precipitate when higher concentrations are used (12, 15).

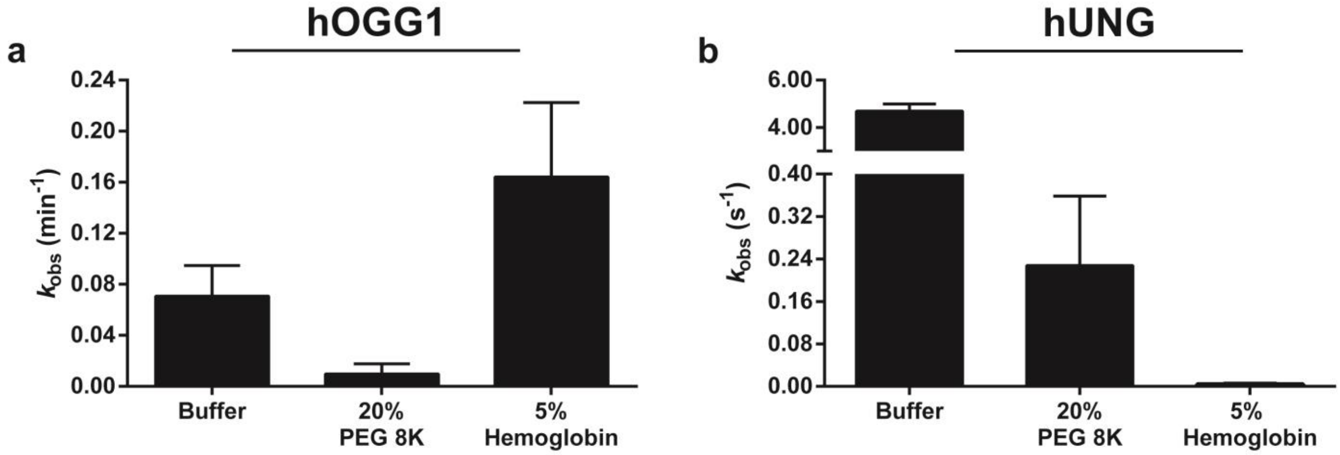

Supplementary Figure S3. Reaction rates in dilute buffer, 20% (w/v) PEG 8K, and 5% (w/v) hemoglobin. **a)** Rates determined by quantifying the amount of product formed as a function upon mixing 1 nM of hOGG1 with 20 nM ^{32}P labeled $S0^{oG}$ 31mer substrate (Buffer: 0.07 ± 0.02 min^{-1}, 20% PEG 8K: 0.010 ± 0.008 min^{-1}, 5% Hemoglobin: 0.16 ± 0.06 min^{-1}). **b)** Reaction rates estimated by determining the rate of total product formation when hUNG was reacted with 40 nM of ^{32}P labeled $S20^{U}$ 90mer substrate in the site transfer assay (**Figures 1 and S4**) (Buffer: 4.7 ± 0.3 s^{-1}, 20% PEG 8K: 0.2 ± 0.1 s^{-1}, 5% Hemoglobin: 0.003 ± 0.001 s^{-1}). Linear reaction rates were measured for both enzymes under conditions of less than 30% product formation.

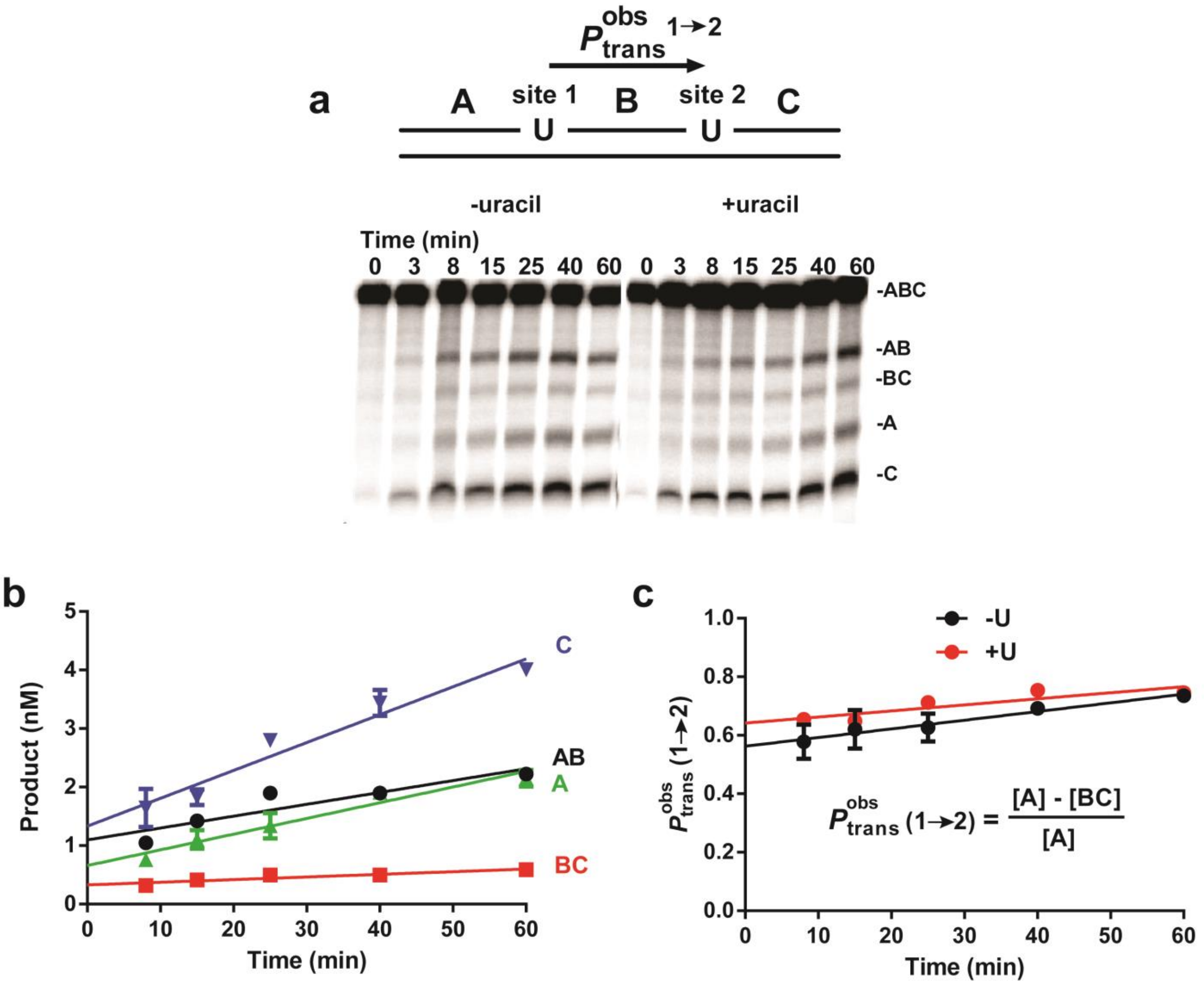

Supplementary Figure S4. Effect of hemoglobin on the site transfer probability (P_{trans}) of hUNG between uracil lesions spaced 20 bp apart. **a)** Phosphorimages of products derived from reaction of hUNG with 40 nM of the 90mer substrate ($S20^U$) in buffer containing 5% hemoglobin in the presence and absence of 20 mM uracil trap. To give reasonable rates, concentrations of hUNG of 500 and 900 pM were used in the absence and presence of the trap. **b)** Velocities of formation of individual fragments derived from $S20^U$ in the absence of uracil trap. Y-intercepts unequal to zero indicate that a burst kinetic phase occurs in the presence of hemoglobin followed by a slower steady-state rate. The data at three minutes time were omitted from this analysis because the signal-to-noise was too small. **c)** Observed probability of directional transfers from site 1 to site 2 in the presence (red) and absence (black) of 20 mM uracil trap. This equation makes the assumption that fragment A is only generated by primary excision events. This assumption is supported by the observation that fragment AB is always present at a high levels indicating poor transfer from site 2→1 (see Supplemental Discussion).

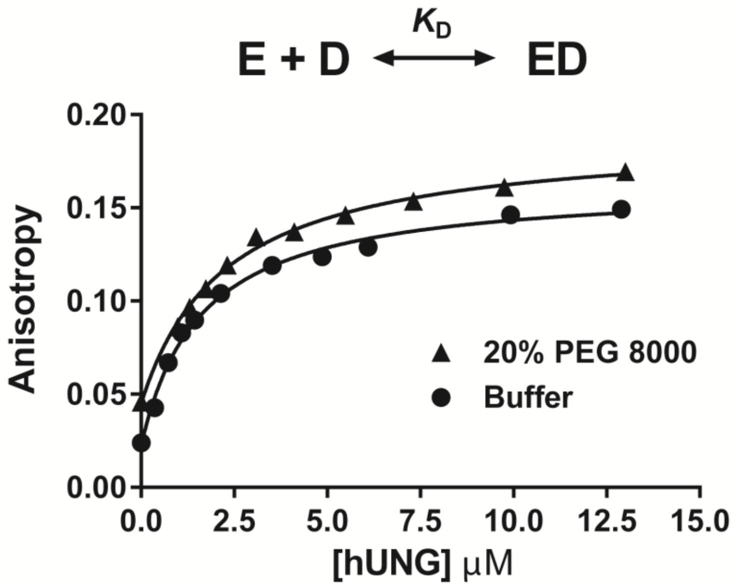

Supplementary Figure S5. Binding affinity (K_D) of hUNG for nonspecific DNA (D^N, 100 nM) in the presence and absence of 20% PEG 8K. The binding affinity was measured by changes in fluorescence anisotropy as a function of hUNG concentration. Inclusion of 20% PEG 8K moderately increases the K_D from 1.3 ± 0.5 μM to 2.3 ± 0.5 μM.

REFERENCES

1. Leipold, M.D., Workman, H., Muller, J.G., Burrows, C.J. and David, S.S. (2003) Recognition and Removal of Oxidized Guanines in Duplex DNA by the Base Excision Repair Enzymes hOGG1, yOGG1, and yOGG2 †. *Biochemistry*, **42**, 11373–11381.

2. Linegar, K.L., Adeniran, A.E., Kostko, A.F. and Anisimov, M.A. (2010) Hydrodynamic radius of polyethylene glycol in solution obtained by dynamic light scattering. *Colloid J*, **72**, 279–281.

3. Devanand, K. and Selser, J.C. (1991) Asymptotic behavior and long-range interactions in aqueous solutions of poly(ethylene oxide). *Macromolecules*, **24**, 5943–5947.

4. Kozer, N., Kuttner, Y.Y., Haran, G. and Schreiber, G. (2007) Protein-protein association in polymer solutions: from dilute to semidilute to concentrated. *Biophysical Journal*, **92**, 2139–2149.

5. Gennes, P.G.D. (1979) Scaling Concepts in Polymer Physics. Cornell University Press, Ithaca, NY.

6. Timchenko, A.A., Kubareva, E.A., Volkov, E.M., Voronina, O.L., Lunin, V.G., Gonchar, D.A., Degtyarev, S.K., Timchenko, M.A., Kihara, H. and Kimura, K. (2006) Structure of Escherichia coli uracil-DNA glycosylase and its complexes with nonhydrolyzable substrate analogues in solution studied by synchrotron small-angle X-ray scattering. *Biophysics*, **51**, 1–7.

7. Schonhoft, J.D. and Stivers, J.T. (2013) DNA translocation by human uracil DNA glycosylase: the case of single-stranded DNA and clustered uracils. *Biochemistry*, **52**, 2536–2544.

8. Parker, J.B., Bianchet, M.A., Krosky, D.J., Friedman, J.I., Amzel, L.M. and Stivers, J.T. (2007) Enzymatic capture of an extrahelical thymine in the search for uracil in DNA. *Nature*, **449**, 433–437.

9. Parikh, S.S., Mol, C.D., Slupphaug, G., Bharati, S., Krokan, H.E. and Tainer, J.A. (1998) Base excision repair initiation revealed by crystal structures and binding kinetics of human uracil-DNA glycosylase with DNA. *EMBO J.*, **17**, 5214–5226.

10. Parikh, S.S., Walcher, G., Jones, G.D., Slupphaug, G., Krokan, H.E., Blackburn, G.M. and Tainer, J.A. (2000) Uracil-DNA glycosylase-DNA substrate and product structures: conformational strain promotes catalytic efficiency by coupled stereoelectronic effects. *Proc. Natl. Acad. Sci. U.S.A.*, **97**, 5083–5088.

11. Cravens, S.L., Hobson, M. and Stivers, J.T. (2014) Electrostatic Properties of Complexes along a DNA Glycosylase Damage Search Pathway. *Biochemistry*, **53**, 7680–7692.

12. Rowland, M.M., Schonhoft, J.D., McKibbin, P.L., David, S.S. and Stivers, J.T. (2014) Microscopic mechanism of DNA damage searching by hOGG1. *Nucleic Acids Res.*, 10.1093/nar/gku621.

13. Krosky, D.J., Song, F. and Stivers, J.T. (2005) The origins of high-affinity enzyme binding to an extrahelical DNA base. *Biochemistry*, **44**, 5949–5959.

14. Ye, Y., Stahley, M.R., Xu, J., Friedman, J.I., Sun, Y., McKnight, J.N., Gray, J.J., Bowman, G.D. and Stivers, J.T. (2012) Enzymatic excision of uracil residues in nucleosomes depends on the local DNA structure and dynamics. *Biochemistry*, **51**, 6028–6038.

15. Schonhoft, J.D. and Stivers, J.T. (2012) Timing facilitated site transfer of an enzyme on DNA. *Nat Chem Biol*, **8**, 205–210.

16. Goins, A.B., Sanabria, H. and Waxham, M.N. (2008) Macromolecular Crowding and Size Effects on Probe Microviscosity. *Biophysical Journal*, **95**, 5362–5373.

17. Georgalis, Y., Philipp, M., Aleksandrova, R. and Krüger, J.K. Light scattering studies on Ficoll PM70 solutions reveal two distinct diffusive modes. *Journal of Colloid and Interface Science*, **386**, 141–147.
